# Photocatalytic ethylene production by oxidative dehydrogenation of ethane with dioxygen on ZnO-supported PdZn intermetallic nanoparticles

**DOI:** 10.1038/s41467-024-45031-6

**Published:** 2024-01-26

**Authors:** Pu Wang, Xingyu Zhang, Run Shi, Jiaqi Zhao, Geoffrey I. N. Waterhouse, Junwang Tang, Tierui Zhang

**Affiliations:** 1 Key Laboratory of Photochemical Conversion and Optoelectronic Materials, Technical Institute of Physics and Chemistry, Chinese Academy of Sciences, Beijing, 100190 China; 2 Center of Materials Science and Optoelectronics Engineering, University of Chinese Academy of Sciences, Beijing, 100049 China; 3 Functional Crystals Lab, Technical Institute of Physics and Chemistry, Chinese Academy of Sciences, Beijing, 100190 China; 4 School of Chemical Sciences, The University of Auckland, Auckland, 1142 New Zealand; 5 Department of Chemical Engineering, University College London, London, WC1E 7JE UK; 6 Industrial Catalysis Center, Department of Chemical Engineering, Tsinghua University, Beijing, 100084 China

**Keywords:** Photocatalysis, Structural properties

## Abstract

The selective oxidative dehydrogenation of ethane (ODHE) is attracting increasing attention as a method for ethylene production. Typically, thermocatalysts operating at high temperatures are needed for C–H activation in ethane. In this study, we describe a low temperature ( < 140 °C) photocatalytic route for ODHE, using O_2_ as the oxidant. A photocatalyst containing PdZn intermetallic nanoparticles supported on ZnO is prepared, affording an ethylene production rate of 46.4 mmol g^–1^ h^–1^ with 92.6% ethylene selectivity under 365 nm irradiation. When we employ a simulated shale gas feed, the photocatalytic ODHE system achieves nearly 20% ethane conversion while maintaining an ethylene selectivity of about 87%. The robust interface between the PdZn intermetallic nanoparticles and ZnO support plays a crucial role in ethane activation through a photo-assisted Mars-van Krevelen mechanism, followed by a rapid lattice oxygen replenishment to complete the reaction cycle. Our findings demonstrate that photocatalytic ODHE is a promising method for alkane-to-alkene conversions under mild conditions.

## Abstract (web summary)

The selective oxidative dehydrogenation of ethane is attracting increasing attention as a method for ethylene production. Here, PdZn supported on ZnO affords record-breaking photocatalytic ethane-to-ethylene conversion rate, emphasizing the pivotal role of the interface between PdZn and ZnO in the process.

## Introduction

Ethylene is one of the most widely used feedstocks in the today’s chemical industry. Currently, high-temperature steam cracking of large hydrocarbons derived from naphtha is the primary method for producing ethylene. The recent discovery of large reserves of shale gas, which contains about 10% ethane (C_2_H_6_), has sparked interest in ethane dehydrogenation as an alternative method for producing ethylene^1^. Researchers are now actively seeking new catalytic materials and technologies capable of efficiently and selectively converting ethane to ethylene.

In recent years, significant progress has been made towards the discovery of efficient catalysts for ethane dehydrogenation, with zeolite-derived and vanadium-based catalysts receiving a lot of attention^2,3^. Given that the C–H bond energy in C_2_H_6_ is exceptionally high (415 kJ mol^–1^), harsh reaction conditions are typically required to achieve the first hydrogen atom extraction from ethane (the rate-limiting step for direct ethane dehydrogenation)^4^. Compared with direct ethane dehydrogenation, the oxidative dehydrogenation of ethane (ODHE), which utilizes an oxidant (e.g. O_2_, CO_2_, or N_2_O), is more thermodynamically favorable (for example, C_2_H_6_  +  0.5O_2_  →  C_2_H_4_  +  H_2_O, ΔG_r_  =  –136.2 kJ mol^–1^) and can deliver both good ethane conversions and acceptable ethylene selectivities^5^. However, thermochemical ODHE still requires high reaction temperatures ( >  500 °C) to achieve a meaningful ethane conversion, stimulating research towards alternative low-temperature ODHE processes powered by sustainable energy sources^6^.

Photocatalysis holds great potential for the solar-driven activation of small molecules such as O_2_, CO_2_, and CH_4_ under mild conditions. Metal oxide-based semiconductor photocatalysts, such as ZnO and TiO_2_, have the ability to generate activated lattice oxygen upon illumination^7,8^, enabling activation of inert chemical bonds via lattice oxygen-mediated photocatalytic oxidative pathways. For examples, Au-ZnO/TiO_2_^7^, Au(Pt, Pd, Ag)/ZnO^9^, and Ag/ZnO^10^ photocatalysts show activity for the photocatalytic methane coupling, partial oxidation, and combustion, respectively. These catalysts all use photogenerated active oxygen species (O^–^) derived from TiO_2_ or ZnO lattice oxygen for C–H bond activation. Moreover, the introduction of metal (metal alloy) nanoparticles with appropriate metal–support interaction boosts interfacial charge transfer and surface active adsorbent conversion, thus speeding up the catalytic reaction kinetics^11^. Tang et al. reported that AuCu−ZnO photocatalyst achieved methane partial oxidation for methanol and formaldehyde production with efficient charge transfer enhanced by Au and Cu species^12^. Whilst photocatalytic C–H bond activation in methane conversion has received considerable attention, no research has yet been conducted on photocatalytic ODHE for ethylene production, motivating a detailed investigation.

Herein, we synthesized PdZn intermetallic nanoparticles supported on ZnO (PdZn-ZnO) as a photocatalyst for ODHE with O_2_. Under 365 nm illumination, a flow photocatalytic system containing PdZn-ZnO delivered a C_2_H_4_ production rate of 46.4 mmol g^–1^ h^–1^ with 92.6% selectivity. This level of performance was vastly superior to that of photocatalysts containing Pd or other intermetallic nanoparticles on ZnO, whilst also outperforming most high-temperature thermochemical ODHE processes. Our comprehensive characterization studies demonstrate that a robust PdZn-ZnO interface effectively enhances the photogeneration of O^–^ (from ZnO lattice oxygen), which in turn activates the C–H bond in ethane. Further, the PdZn-ZnO metal-support interaction allows fast electron transfer for efficient dioxygen reduction and ZnO lattice oxygen replenishment. The combination of these processes significantly reduced the apparent activation energy for ODHE to only 18.4 kJ mol^–1^, with PdZn-ZnO delivering an ethane conversion of nearly 20% with about 87% selectivity in simulated shale gas and showing feasibilities in the selective oxidative dehydrogenation of propane and butane. Results identify photocatalysis as a promising strategy for the selective production of ethylene from ethane at low temperatures.

## Results and Discussion

### Synthesis and characterization of PdZn-ZnO photocatalysts

**Fig. 1:**
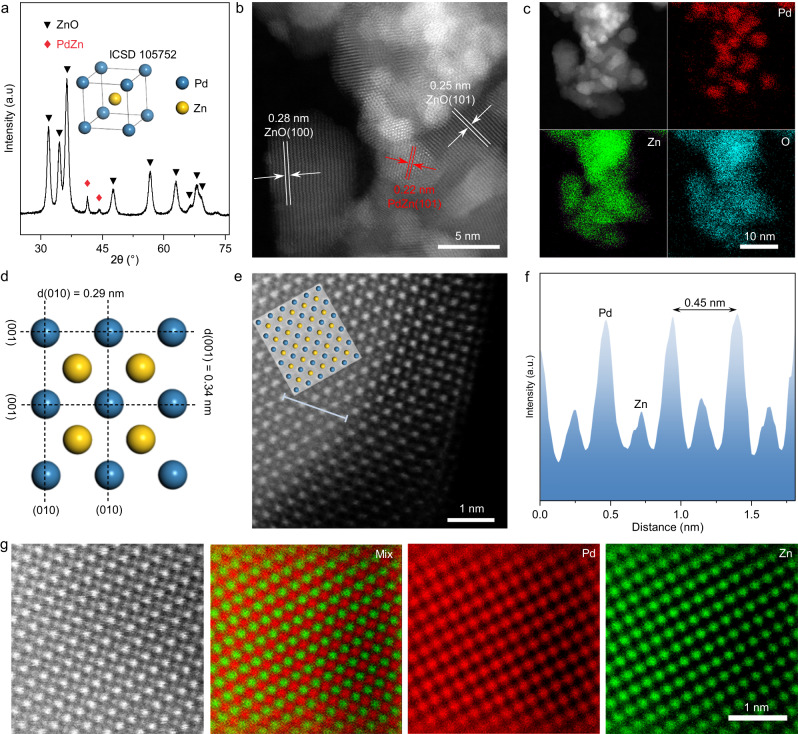
Structural characterization of PdZn-ZnO. **a** XRD patterns. Inset: crystal structure of PdZn. **b** HAADF-STEM image. **c** Energy-dispersive X-ray element maps. **d** Projection in the [010] direction for tetragonal PdZn. The yellow and blue spheres represent Zn and Pd atoms, respectively. **e** Aberration-corrected HAADF-STEM image. Inset: schematic of the PdZn intermetallic structure. **f** Intensity profiles measured from (**e**). **g** Aberration-corrected HAADF-STEM image and corresponding atomic-resolution elemental maps.

The X-ray diffraction (XRD) pattern of the Pd-doped Zn_5_(CO_3_)_2_(OH)_6_ precursor is shown in Supplementary Fig. 1. All peaks can be assigned to monoclinic hydrozincite, Zn_5_(CO_3_)_2_(OH)_6_ (JCPDS No. 19-1458)^13^. No peaks were seen for any Pd-containing species, indicating that Pd was highly dispersed in the sample^14^. Transmission electron microscopy (TEM) revealed Pd-doped Zn_5_(CO_3_)_2_(OH)_6_ possessed a nanosheet-like structure, with energy dispersive X-ray spectroscopy (EDS) confirming that Zn, O, and Pd were uniformly dispersed in the sample (Supplementary Fig. 2). After annealing at 300 °C in a H_2_ flow (10 vol.% H_2_ in Ar) for 4 h, the precursor was transformed into a PdZn-ZnO catalyst. As shown in the XRD pattern (Fig. 1a), the PdZn-ZnO catalyst consists of hexagonal wurtzite ZnO (JCPDS No. 36-1451) and tetragonal intermetallic PdZn nanoparticles (JCPDS No. 06-0620)^15,16^ After dissolving the ZnO support with dilute hydrochloric acid, the isolated PdZn nanoparticles show a XRD pattern matched well with the predicted PdZn intermetallic nanoparticle (Supplementary Fig. 3). The Pd loading in PdZn-ZnO was determined to be 1.57 wt.%, which was close to the expected nominal value (2.0 wt.%) (Supplementary Table 1). The high-angle annular dark-field scanning TEM (HAADF-STEM) image and EDS element maps confirmed the presence of PdZn intermetallic nanoparticles dispersed on ZnO nanoparticles (Fig. 1b, c). Lattice fringes with spacings of 0.28, 0.25, and 0.22 nm corresponded to ZnO (100), ZnO (101) and PdZn (101) planes, respectively^17,18^. For comparison, photocatalysts consisting of pristine ZnO and ZnO-supported Pd nanoparticles (Pd-ZnO) were prepared. The structure, morphology, and specific surface area of ZnO and Pd-ZnO photocatalysts were similar to that of PdZn-ZnO (Supplementary Fig. 4 to 6).

The (100) crystal plane of PdZn shows an interphase arrangement of Pd and Zn atomic columns and contains of (001) and (010) planes (Fig. 1d). The aberration-corrected HAADF-STEM image of a PdZn nanoparticle (Fig. 1e) in PdZn-ZnO shows highly ordered rectangular arrays with alternating bright and dark columns of atoms. The fast Fourier-transform (FFT) pattern is consistent with the simulation results for a PdZn intermetallic nanoparticle in the [010] direction (Supplementary Fig. 7). The corresponding intensity profile presents a periodic oscillation pattern in two perpendicular directions corresponding to [001] (Fig. 1f). The regular atomic configurations of Pd and Zn atoms in the PdZn nanoparticle were also evident in the corresponding elemental maps (Fig. 1g), further confirming the presence of PdZn intermetallic nanoparticles in PdZn-ZnO.

### Photocatalytic ODHE performance evaluation

**Fig. 2:**
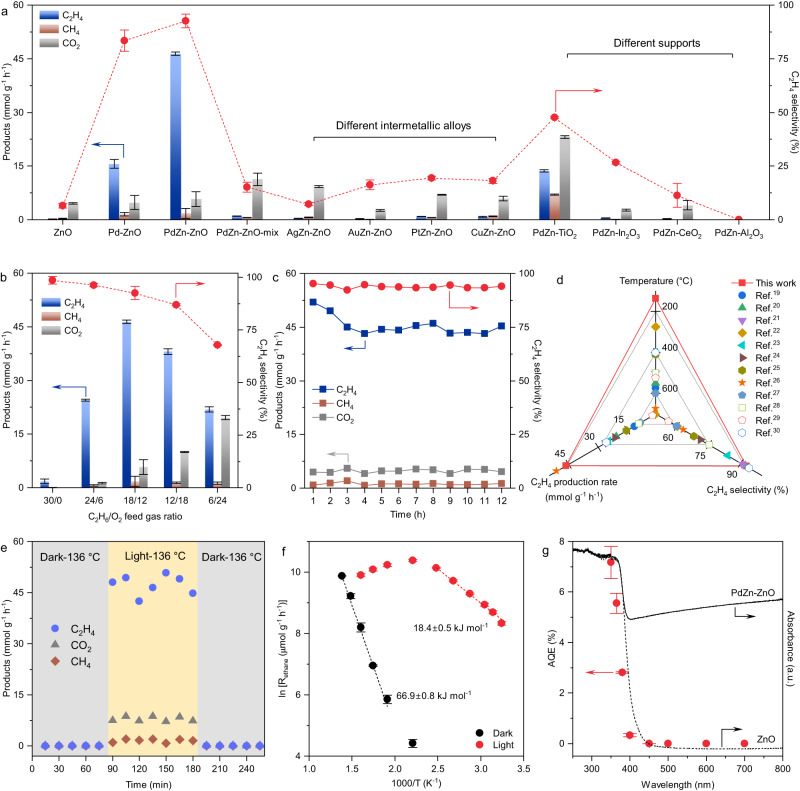
Photocatalytic ODHE performance of PdZn-ZnO. **a** ODHE tests over PdZn-ZnO and comparison photocatalysts. Reaction condition: 5.0 mg photocatalyst, C_2_H_6_ (5 vol.% in Ar, 18 mL min^–1^)  +  O_2_ (1 vol.% in Ar, 12 mL min^–1^), 365 nm LED, 600 mW cm^–2^. **b** Photocatalytic activity at a total flow rate of 30 mL min^–1^ with different C_2_H_6_/O_2_ feed gas ratios. **c** Photocatalytic stability test for 12 h of continuous irradiation. **d** Performance comparison with reported thermocatalysts. **e** Photocatalytic activity at 136 °C under dark-light switch conditions. **f** Arrhenius plots measured in the dark and light conditions. Light intensity = 55.9 mW cm^–2^. **g** Wavelength-dependent AQE and diffuse reflectance spectra of ZnO and PdZn-ZnO. Error bars represent standard deviations obtained from three independent measurements.

Photocatalytic ODHE tests were carried out using a home-made continuous flow photoreactor equipped with a 365 nm LED lamp as the light source (see Methods for experimental details). A mixture of C_2_H_6_ (5 vol.% in Ar, 18 mL min^–1^) and O_2_ (1 vol.% in Ar, 12 mL min^–1^) with a total flow rate of 30 mL min^–1^ was introduced as the feed gas. Figure 2a and Supplementary Table 2 show the photocatalytic C_2_H_4_ production rate and C_2_H_4_ selectivity for PdZn-ZnO and a series of reference samples. PdZn-ZnO exhibited a C_2_H_4_ production rate of 46.4 mmol g^–1^ h^–1^ with an ethylene selectivity of 92.6%. No liquid carbon-containing products, such as methanol and ethanol, were formed during the photocatalytic reaction (Supplementary Fig. 8). The optimal photocatalyst dosage and nominal Pd loading were determined to be 5.0 mg and 2.0 wt.%, respectively (Supplementary Fig. 9 to 11). The calcination temperature of the precursor was optimized to be 300 °C (Supplementary Fig. 12 and 13). The Pd-ZnO reference delivered a C_2_H_4_ production rate of 15.6 mmol g^–1^ h^–1^ with 83.4% ethylene selectivity, which is significantly lower than PdZn-ZnO. It should be noted that for pristine ZnO, only CO_2_ and traces of C_2_H_4_ were generated, with no obvious enhancement observed for a physical mixture of ZnO with PdZn nanoparticles (denoted as PdZn-ZnO-mix, Supplementary Fig. 14). We also prepared other intermetallic nanoparticles (AgZn, AuZn, PtZn, CuZn) supported on ZnO (denoted as MZn-ZnO, M = Ag, Au, Pt, and Cu) using methods similar to that used to prepare PdZn-ZnO (Supplementary Fig. 15). However, CO_2_ was the major product for all these photocatalysts. In addition, catalysts containing PdZn nanoparticles on other metal oxide supports, including TiO_2_, In_2_O_3_, CeO_2_, and Al_2_O_3_ were prepared (Supplementary Fig. 16). PdZn-TiO_2_ prefer to over-oxidize C_2_H_6_ to CO_2_ with a low C_2_H_4_ selectivity of 47.6%. PdZn-In_2_O_3_, PdZn-CeO_2_, and PdZn-Al_2_O_3_ showed negligible activity under the same reaction conditions. Clearly, the ZnO support actively participated in photocatalytic ODHE over PdZn-ZnO. Above results indicated that PdZn intermetallic nanoparticles, ZnO, and the interfacial contact between PdZn and ZnO were indispensable to the outstanding photocatalytic performance.

Figure 2b shows that the C_2_H_6_/O_2_ ratio had a strong influence on the activity and selectivity of PdZn-ZnO for ethane-to-ethylene conversion. The C_2_H_4_ selectivity decreased from nearly 100% to 67.7% as the C_2_H_6_ (5 vol.% in Ar)/O_2_ (1 vol.%  in Ar) feed gas ratio was changed from 30/0 to 6/24 (total flow rate = 30 mL min^–1^). The production rate of C_2_H_4_ followed a volcano-type relationship, with the highest rate achieved at a feed gas ratio of 18/12. The effect of the total flow rate was then investigated at the C_2_H_6_/O_2_ ratio of 18/12 (Supplementary Fig. 17). The C_2_H_4_ production rate increased gradually from 5 mL min^–1^ to 30 mL min^–1^, reaching a maximum of 46.4 mmol g^–1^ h^–1^. The selectivity to C_2_H_4_ remained above 92% under these flow conditions. For a batch reactor (flow rate = 0), the best performance was realized after about 180 s of irradiation (15.0% C_2_H_6_ conversion, 6579.3 μmol g^–1^ C_2_H_4_ production, and 85% ethylene selectivity, Supplementary Fig. 18), with the short time to reach the optimum being due to depletion of O_2_ in the batch reactor. At higher reactant concentrations, the ethylene production rate exhibited a substantial increase, reaching up to 225.9 mmol g^–1^ h^–1^. Notably, the ethylene selectivity showed a gradual decline from 92.6% to 61.9% as the concentration of reactants increased (Supplementary Fig. 19). This suggests the importance of finding a balance between the rate of product formation and selectivity. During a 12 h of continuous photocatalytic stability test under the optimized reaction condition, the production rate of C_2_H_4_ over PdZn-ZnO fluctuated around 45 mmol g^–1^ h^–1^ with no obvious deactivation and a stable ethylene selectivity of around 92% (Fig. 2c). The spent catalyst showed almost no structural changes as confirmed by XRD (Supplementary Fig. 20). The nearly 100% carbon balance, supported by thermogravimetric and Raman spectroscopy analyses, confirms the absence of carbon deposition (Supplementary Fig. 21-23).

As shown in Fig. 2d and Supplementary Table 3, the photocatalytic ODHE performance of PdZn-ZnO was superior to representative thermocatalysts reported to date when considering the three main reaction parameters: temperature, C_2_H_4_ production rate, and selectivity^19–30^. Moreover, the as-developed PdZn-ZnO photocatalytic system also demonstrated exceptional performance for the oxidative dehydrogenation of propane (a propene production rate of 54.0 mmol g^–1^ h^–1^ with 89.4% propene selectivity) and butane (a butene production rate of 59.1 mmol g^–1^ h^–1^ with 95.9% butene selectivity) (Supplementary Fig. 24). Additionally, by replacing the ethane feed gas with a simulated shale gas (45 vol.% CH_4_, 5 vol.% C_2_H_6_, balanced with Ar), a C_2_H_4_ production rate of about 60 mmol g^–1^ h^–1^ was achieved, with about 87% ethylene selectivity at nearly 20% ethane conversion (Supplementary Fig. 25).

It is noteworthy that the surface temperature of the PdZn-ZnO photocatalyst increased under light irradiation due to the photothermal effects, and reached 136 °C at 600 mW cm^–2^. (Supplementary Fig. 26a). As depicted in Fig. 2e, no significant product formation was observed by electrically heating the reactor containing PdZn-ZnO to 136 °C in the dark. However, on exposing the photocatalyst to light irradiation, the C_2_H_4_ production rate was greatly enhanced at the same temperature, and showed a linear relationship with the light intensity (Supplementary Fig. 26b). The results indicate that the ethane oxidative dehydrogenation process involved a photocatalytic mechanism. Arrhenius plots based on ethane reaction rates (*R*_ethane_) at different temperatures are shown in Fig. 2g. The apparent activation energy for ODHE over PdZn-ZnO under light conditions (18.4 kJ mol^–1^) was significantly lower than in the dark (66.9 kJ mol^–1^), implying that photons changed the reaction pathway for ODHE and significantly reduced the reaction energy barrier. *R*_ethane_ values decreased under light conditions at temperatures greater than 180 °C, which is explained by shortened lifetime of photogenerated charge carriers and increased oxygen consumption due to ethane overoxidation at such high temperatures (Supplementary Fig. 27 and 28)^31^. The wavelength-dependent apparent quantum efficiency (AQE) for photocatalytic ODHE over PdZn-ZnO was found to be 7.2%, 5.5%, 2.8%, and 0.3% under monochromatic irradiation at 350, 365, 380, and 400 nm, respectively (Fig. 2f). This trend closely matched the absorbance spectrum of ZnO, indicating that photo-excitation of ZnO was a key process in ethylene production.

### Mechanistic investigations

**Fig. 3:**
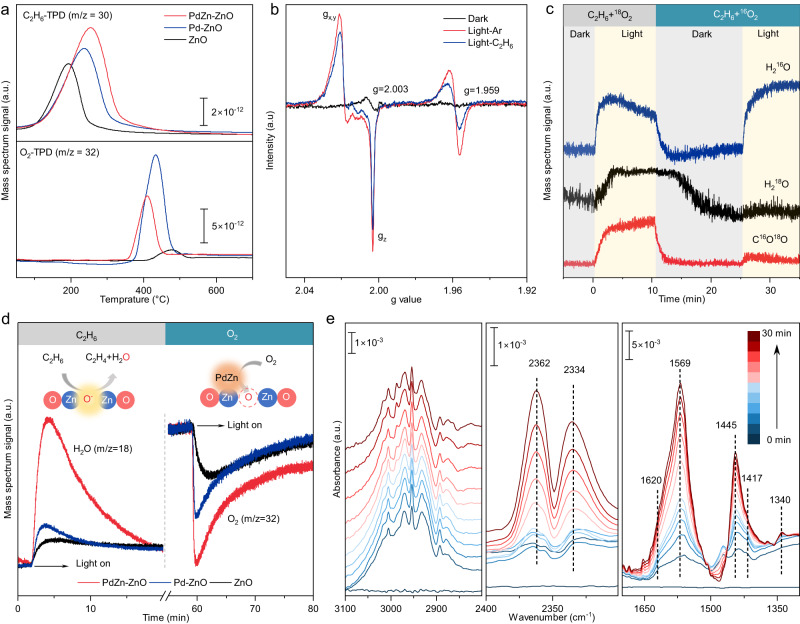
Spectral studies for reaction intermediates of photocatalytic ODHE. **a** C_2_H_6_-TPD and O_2_-TPD spectra for ZnO, Pd-ZnO and PdZn-ZnO. **b** In situ EPR spectra for ZnO exposed to Ar in the dark and under 365 nm irradiation, and exposed to C_2_H_6_  under 365 nm irradiation. **c** Evolution of reactants and products during ODHE monitored by online MS. **d** Time-resolved online MS data for C_2_H_6_ dehydrogenation over ZnO, Pd-ZnO and PdZn-ZnO. **e** In situ FT-IR spectra collected from PdZn-ZnO in a feed gas (3 vol.% C_2_H_6_ + 0.4 vol.% O_2_, balanced with Ar) at different irradiation times.

PdZn-ZnO and two reference samples, ZnO and Pd-ZnO, were selected for further mechanistic investigations. Firstly, temperature-programmed desorption experiments for C_2_H_6_ and O_2_ (C_2_H_6_-TPD and O_2_-TPD) were conducted, with the results shown in Fig. 3a. The data show that C_2_H_6_ (m/z = 30) desorbed at 190.7, 233.8 and 254.6 °C on ZnO, Pd-ZnO, and PdZn-ZnO, respectively. Clearly, PdZn-ZnO shows a higher desorption temperature than the other samples, indicating a strong chemical adsorption towards C_2_H_6_. Furthermore, PdZn-ZnO demonstrates the highest ethane adsorption strength when compared to PdZn-TiO_2_, PdZn-In_2_O_3_, PdZn-CeO_2_, and PdZn-Al_2_O_3_ (Supplementary Fig. 29). This heightened ethane adsorption capability could be a key factor contributing to the significantly improved photocatalytic ODHE performance observed in PdZn-ZnO compared to its counterparts. In the O_2_-TPD profiles, O_2_ (m/z = 32) desorption signals were observed between 400 and 500 °C for all three samples, which can be ascribed to the desorption of chemisorbed oxygen species. The O_2_ adsorption capacity of pristine ZnO was modest, and largely attributable to O_2_ adsorption/dissociation on oxygen defects produced by sample pretreatment in a hydrogen atmosphere^32^. Once Pd or PdZn nanoparticles were introduced, the amount of adsorbed oxygen increased significantly, indicating that Pd and PdZn were the dominant active sites for O_2_ adsorption and activation^33^.

The efficient separation and transport of photogenerated charge carriers are critical for photocatalytic reactions^34^. The photoluminescence (PL) intensity under 365 nm excitation was notably decreased for Pd-ZnO and PdZn-ZnO compared with pristine ZnO, indicating improved separation of photo-excited electron-hole pairs (Supplementary Fig. 30). To investigate the carrier dynamics further, time-resolved transient absorption (TA) spectra were then recorded under 365 nm excitation and probed at 520 nm (Supplementary Fig. 31). The results showed that PdZn-ZnO had the longest lifetime of photogenerated charge carriers (187.3 ps), compared to Pd-ZnO (132.9 ps) and ZnO (118.6 ps), confirming its superior carrier separation efficiency. Next, in situ electron paramagnetic resonance (EPR) experiments were conducted to investigate the electron transfer and ethane activation processes. As shown in Fig. 3b and Supplementary Fig. 32, under dark conditions exposed to Ar atmosphere, both ZnO and PdZn-ZnO showed a symmetric EPR peak with a g value of 2.003, which could be attributed to unpaired electrons trapped at oxygen vacancies (O_v_) in ZnO^35^. Under light irradiation, a symmetric EPR peak at g = 1.959 appeared for ZnO due to trapped photogenerated electrons (i.e. Zn^+^ species)^36^. However, this trapped electron signal was weak for PdZn-ZnO, suggesting that the excited electrons in ZnO were transferred to PdZn nanoparticles, which acted as electron acceptors^37^. Additionally, anisotropic EPR signals, namely g_*x*_ = 2.023, g_*y*_ = 2.019, and g_*z*_ = 2.003, also appeared under irradiation, which can be assigned to O^–^ species created by the ZnO lattice oxygen (formally O^2–^) trapping photogenerated holes^38^. After switching the contacting atmosphere from Ar to C_2_H_6_ under light conditions, the intensity of the EPR signal related to O^–^ species decreased significantly for both ZnO and PdZn-ZnO, inferring the reaction of surface O^–^ with C_2_H_6_ molecules. The results provide strong evidence for enhanced interfacial electron transfer from ZnO to PdZn in PdZn-ZnO, whilst also establishing the crucial role of photogenerated surface O^–^ derived from lattice oxygen in C_2_H_6_ activation.

Oxygen isotope labeling tracing experiments were utilized to explore the key role of lattice oxygen in photocatalytic ODHE. Figure 3c shows the results of the ^18^O isotope experiments, with online mass spectrometry (MS) used to detect ^18^O-containing products of ODHE (see Methods for more details). Firstly, a gas mixture containing C_2_H_6_ and ^18^O_2_ was fed to the reactor in the dark to remove residual gas impurities in the flow chamber. Upon irradiation of PdZn-ZnO, strong signals due to H_2_^16^O and C^16^O^18^O were observed confirming that surface lattice oxygen (^16^O) on ZnO was an active site for water production and ethane conversion during photocatalytic ODHE. This result was further corroborated by the ^18^O-tracer experiments (Supplementary Fig. 33), in which ^18^O had been pre-incorporated in the ZnO lattice of PdZn-ZnO. After 10 min of illumination, the light was turned-off and the atmosphere switched to a gas mixture of C_2_H_6_ and ^16^O_2_. The detection of ^18^O in the oxygen-containing gas products (H_2_^18^O, C^16^O^18^O) confirmed that ^18^O from the ^18^O_2_ feed replenished O_v_ during the first irradiation period and could participate in the following ODHE reaction. This lattice oxygen replenishment process was also confirmed by the light-induced oxygen isotope exchange effect between ^18^O_2_ and PdZn-ZnO (Supplementary Fig. 34).

Time-resolved online MS spectra for C_2_H_6_ dehydrogenation over PdZn-ZnO, Pd-ZnO, and ZnO were then collected (Fig. 3d). An ethane gas flow was first introduced into the reactor, with a H_2_O signal being detected for all photocatalysts under light irradiation as a result of lattice oxygen consumption and O_v_ generation on ZnO during the ODHE reaction. The light was then turned off and the atmosphere switched to O_2_ for a period of time. When the light was again turned on, the signal for O_2_ in the gas phase decayed, indicating the consumption of O_2_ to fill O_v_ sites. PdZn-ZnO showed the highest water generation and dioxygen consumption signals under illumination, indicating a significantly promoted lattice oxygen-mediated photocatalytic ODHE through a photo-enhanced Mars-van Krevelen (M-K) pathway. That is, the photogenerated surface O^–^ from lattice oxygen on ZnO extracts hydrogen atoms from ethane, resulting in the formation of water molecules and O_v_. Then, O_2_ adsorbed on PdZn is activated by photo-excited electrons, with the O_2_ molecule dissociating to fill O_v_ and complete the reaction cycle.

In situ Fourier transform infrared (FT-IR) spectra were collected to gain additional information about the reaction intermediates and reaction mechanism (see Methods for experiment details). As shown in Fig. 3e, a series of absorption peaks appeared over PdZn-ZnO in a feed gas (3 vol.% C_2_H_6_ + 0.4 vol.% O_2_, balanced with Ar) under light conditions, with the intensities of the peaks gradually increasing with irradiation time. A Zn–OH bending vibration at 1417 cm^–1^ was detected^39^, indicating the presence of surface-adsorbed hydroxyl groups (*OH) generated by hydrogen atom extraction from ethane on surface lattice oxygen (i.e. photo-generated O^–^ sites) of ZnO^40^. The absorption peaks at 1445/1620 cm^–1^, together with the absorption band around 2950 cm^–1^, could be assigned to C = C and C–H stretching vibrations of adsorbed C_2_ intermediates (*C_2_H_4+n_, n = 0, 1, 2)^41,42^. The peaks at 1569 cm^–1^ and 1340 cm^–1^ were assigned to adsorbed carboxyl species (HCOO*) species associated with ethane overoxidation products, which were also evidences by gaseous CO_2_ peaks at 2334 cm^–1^ and 2362 cm^–1^ ^43^. The FT-IR results demonstrate the formation of surface-adsorbed *OH, *C_2_H_4+n_, and HCOO* during ODHE over PdZn-ZnO. These are the expected intermediates for lattice oxygen-mediated photocatalytic ODHE (along with some minor intermediates and products associated with the over-oxidation of ethane).

**Fig. 4:**
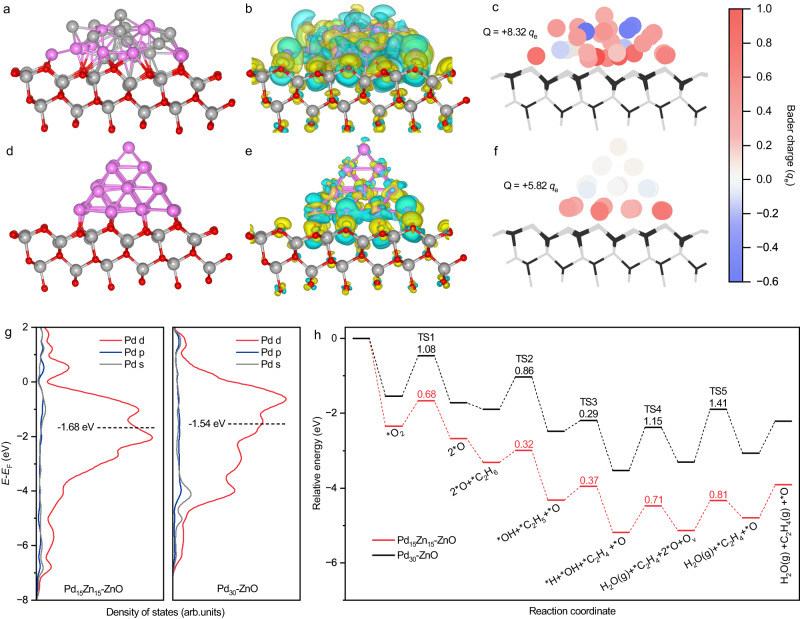
DFT calculations exploring surface charge distributions and reaction kinetics. **a** and **d** Model structures for Pd_15_Zn_15_-ZnO (**a**) and Pd_30_-ZnO (**d**). **b** and **e** Charge density difference plots for Pd_15_Zn_15_-ZnO (**b**) and Pd_30_-ZnO (**e**). The Pd, Zn, and O atoms are shown in purple, gray, and red. The yellow and cyan surfaces correspond to regions of charge gain (accumulation) and loss (depletion). The isovalue of the isosurfaces is 3.0 × e Å^–3^. (**c** and **f**) Bader charge distributions for Pd_15_Zn_15_-ZnO (**c**) and Pd_30_-ZnO (**f**). The red and blue spheres indicate the extent of charge depletion (positive) and accumulation (negative). **g** Density of states of Pd atoms in Pd_15_Zn_15_-ZnO and Pd_30_-ZnO. The horizontal dashed lines indicate the calculated d-band center. *E*-*E*_F_ represents the energy relative to the Fermi energy level. **h** Calculated potential energy diagrams for ODHE to C_2_H_4_ on Pd_15_Zn_15_-ZnO and Pd_30_-ZnO.

To gain a better understanding of the electron transfer processes and reaction transition states (TS) occurring at the interface between the metal nanoparticle (PdZn or Pd) and the ZnO support, density functional theory (DFT) calculations were performed. Two models were built and optimized to simulate the structure of PdZn-ZnO and Pd-ZnO. A Pd_15_Zn_15_ cluster supported on ZnO(101) (denoted as Pd_15_Zn_15_-ZnO) was used to represent PdZn-ZnO, whilst a Pd_30_ cluster supported on ZnO(101) (denoted as Pd_30_-ZnO) was used to represent Pd-ZnO, as shown in Fig. 4a, d. A charge density difference comparison between the two models revealed that charge redistribution is localized in a limited region near the interface between metal nanoparticles and ZnO supports (Fig. 4b, e). Bader charge analysis predicted increased charge accumulation ( + 8.32 *q*_e_) on the Pd_15_Zn_15_ cluster (Fig. 4c) compared to the Pd_30_ cluster ( + 5.82 *q*_e_, Fig. 4f), indicating that electron transfer from PdZn to ZnO was enhanced compared to electron transfer from Pd to ZnO. The higher binding energy of the Pd 3d signal in PdZn-ZnO than in Pd-ZnO also signifies a more pronounced electron deficiency state associated with interfacial electron transfer (Supplementary Fig. 35). DFT calculations also showed that the strong interfacial electron delocalization and transfer in Pd_15_Zn_15_-ZnO resulted in a more negative Pd d-band center position (–1.68 eV) compared with Pd_30_-ZnO (–1.54 eV) (Fig. 4g). The more negative d-band center in the case of supported PdZn contributes to a downshifted antibonding state for oxygen adsorbates, leading to an increased d orbital occupancy and weakened adsorption energy of activated oxygen adsorbates^44,45^. This was expected to be beneficial for subsequent lattice oxygen replenishment processes on adjacent ZnO during ODHE.

The reaction kinetics of dioxygen activation and ethane dehydrogenation to ethylene over Pd_15_Zn_15_-ZnO and Pd_30_-ZnO were then simulated, and the energy profiles are shown in Fig. 4h. Additional information on the models and energy values are summarized in Supplementary Fig. 36-37 and Supplementary Table 4-5. The adsorption energy of O_2_ on Pd_15_Zn_15_-ZnO was found to be significantly higher (–2.35 eV) than that on Pd_30_-ZnO (–1.54 eV). The adsorbed oxygen (*O_2_) then dissociates to two oxygen atoms (*O_2_  →  2*O, TS1), with activation barriers (*E*_a_) of 0.68 eV and 1.08 eV on Pd_15_Zn_15_-ZnO and Pd_30_-ZnO, respectively. These results are consistent with the rapid oxygen consumption over PdZn-ZnO observed on the time-resolved online MS (Fig. 3d).

Pd_15_Zn_15_-ZnO was found to have an *E*_a_ of 0.32 eV for *O-assisted C–H bond scission in C_2_H_6_ (*O  +  *C_2_H_6_  →  *C_2_H_5_  +  *OH, TS2), which was 0.54 eV lower than that of Pd_30_-ZnO, indicating that ethane activation was kinetically favored over PdZn-ZnO. Subsequently, the β-H of *C_2_H_5_ is extracted to form *C_2_H_4_ and *H on ZnO (TS3). Then, *OH and *H combine to form H_2_O (TS4) via two possible pathways, with the oxygen in H_2_O coming from either surface lattice oxygen (M-K pathway) or dissociated dioxygen (Langmuir-Hinshelwood, L-H pathway). The barriers of these two pathways over Pd_15_Zn_15_-ZnO were calculated to be 0.71 eV and 0.92 eV, respectively (Supplementary Fig. 38 and 39), suggesting that the M-K pathway is kinetically favored. The M-K pathway leaves an O_v_ on ZnO and an additional *O on PdZn, respectively, followed by a ZnO lattice oxygen replenishment process between the two species (TS5) with an *E*_a_ of 0.81 eV. Pd_30_-ZnO exhibits larger *E*_a_ for both TS4 and TS5 compared with Pd_15_Zn_15_-ZnO (Supplementary Fig. 40 and 41). We compared the energy profiles of the first two reaction steps (TS1 and TS2 as potential rate-determining steps) with models constructed for other Pd-based intermetallic nanoparticles on ZnO (Pd_15_In_15_-ZnO, Pd_15_Cu_15_-ZnO and Pd_15_Ni_15_-ZnO). Results showed that Pd_15_Zn_15_-ZnO exhibited the lowest *E*_a_ for both oxygen activation and hydrogen extraction from ethane, validating its outstanding photocatalytic ODHE performance (Supplementary Fig. 42 and 43).

**Fig. 5:**
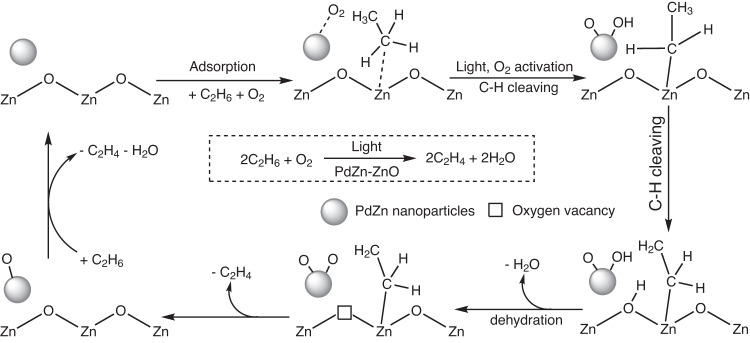
Proposed reaction mechanism for photocatalytic ODHE over PdZn-ZnO. Bond angles and bond lengths are not accurate in the schematic. The gray sphere represents a PdZn nanoparticle.

Based on the results presented, we propose a probable reaction mechanism for the selective photocatalytic ODHE over PdZn-ZnO (Fig. 5). Initially, photoexcited electron-hole pairs are generated in ZnO under UV (365 nm) irradiation. The photogenerated electrons in ZnO are efficiently transferred to PdZn nanoparticles, activating O_2_ to adsorbed oxygen atoms. Meanwhile, the photogenerated holes reaching the surface lattice oxygen of ZnO to form O^–^, which activates C–H bond scission in C_2_H_6_ to form *C_2_H_5_ and *OH. Subsequently, β-H cleavage in *C_2_H_5_ leads to the formation of *C_2_H_4_ and a water molecule, both of which desorb from the photocatalysts (leaving an O_v_ on the ZnO surface). Finally, the O_v_ is filled by the adsorbed oxygen atom spilling over from the PdZn sites.

In summary, we report the first example of selective photocatalytic ODHE with O_2_ for ethylene production. A PdZn-ZnO photocatalyst, containing PdZn intermetallic nanoparticles, was prepared via thermal hydrogen reduction of a Pd-doped Zn_5_(CO_3_)_2_(OH)_6_ precursor. The strong electron transfer interactions at the PdZn-ZnO interface promotes the photogeneration of surface O^–^ active sites on ZnO to activate the C–H bonds in ethane, leading to the formation of ethylene and water. Photo-excited electron transfer from ZnO to PdZn facilitated dioxygen dissociation, allowing fast replenishment of ZnO lattice oxygen consumed in the Mars-van Krevelen mechanism. Photocatalytic ODHE on PdZn-ZnO had an apparent activation energy of 18.4 kJ mol^–1^ under irradiation conditions, much lower than that in the dark (66.9 kJ mol^–1^). The PdZn-ZnO photocatalyst shows an ethylene production rate of 46.4 mmol g^–1^ h^–1^ with 92.6% selectivity at low temperatures, exhibiting high alkene production rates and selectivity for photocatalytic dehydrogenation of propane and butane, as well as ethane in simulated shale gas. This work offers a proof-of-concept investigation, demonstrating that low-temperature photocatalytic ODHE is a promising new approach for ethylene production from ethane-containing feedstocks. Future work should be directed towards more detailed investigation of reaction mechanisms and also reactor designs for process scale-up.

## Methods

### Chemicals

Zn(NO_3_)_2_·6H_2_O, Na_2_CO_3_ and Cu(NO_3_)_2_·3H_2_O were obtained from Beijing Innochem Science & Technology Co., Ltd. Na_2_PdCl_4_, H_2_PtCl_6_, AgNO_3_, HAuCl_4_ were purchased from Shanghai Aladdin Biochemical Technology Co., Ltd. C_2_H_6_ (5 vol.% in Ar), O_2_ (1 vol.% in Ar) and a mixed reactant gas (3 vol.% C_2_H_6_, 1 vol.% O_2_, balanced with Ar) were purchased from Beijing Beiyang United Gas Co., Ltd. ^18^O isotopically labeled oxygen (1 vol.% ^18^O_2_ in Ar) was obtained from Shanghai Maotoo Specialty Gases Co., Ltd. All materials were used without further purification. Deionized water was used in the synthesis of all catalysts.

### Preparation of catalysts

- PdZn-ZnO. Pd-doped Zn_5_(CO_3_)_2_(OH)_6_ precursor (Pd-Zn_5_(CO_3_)_2_(OH)_6_) was synthesized according a commonly used coprecipitation method. Typically, for the preparation of the PdZn-ZnO photocatalyst, 2.97 g Zn(NO_3_)_2_·6H_2_O and a certain amount of Na_2_PdCl_4_ were dissolved in deionized water. Then, a Na_2_CO_3_ aqueous solution was added dropwise to the metal salt solution under continuous stirring. Next, the Pd-Zn_5_(CO_3_)_2_(OH)_6_ product was collected by centrifugation, washed several times with deionized water and then finally vacuum freeze-dried for 24 h. Next, the Pd-Zn_5_(CO_3_)_2_(OH)_6_ precursor was heated at 5 °C min^–1^ to 300 °C under a H_2_/Ar (10/90) flow (300 mL min^–1^), then held at 300 ^o^C for 4 h.
- MZn-ZnO. The synthesis methods for AuZn-ZnO, AgZn-ZnO, PtZn-ZnO, CuZn-ZnO photocatalysts (with the mass percentage of Au, Ag, Pt or Cu 2 wt.% relative to ZnO), were similar to those described for the PdZn-ZnO photocatalyst with different metals salts being used as required.
- ZnO and Pd-ZnO. Pristine ZnO photocatalyst was prepared by heating Zn_5_(CO_3_)_2_(OH)_6_ at 300 °C in a H_2_/Ar (10/90) flow (300 mL min^–1^) for 4 h. The Pd-ZnO photocatalysts was synthesized using a NaBH_4_ reduction method^46^ with the above synthesized ZnO as raw material, followed by heating in a nitrogen gas stream at 300 °C for 4 h.
- PdZn-ZnO-mix. For the synthesis of PdZn-ZnO-mix sample, 1.0 g of PdZn-ZnO was immersed in 30 mL of an aqueous 1 mol L^–1^ HCl solution for 10 min to dissolve the ZnO support, then collected by centrifugation and washed with water. The obtained PdZn nanoparticles was dispersed in 100 mL of deionized water together with 980 mg of ZnO. The PdZn and ZnO dispersion was sonicated for 30 min, followed by freeze-dried for 24 h.

### Characterizations

The structure and crystallinity of the photocatalysts were examined by X-ray diffraction (XRD, Bruker AXSD8 Advance, Germany). The diffractometer was equipped with a Cu Kα radiation source (*λ*  =  1.5405 Å) operating at 40 kV. Morphologies and structure of the photocatalysts were studied using TEM (JEM, 2100 F, Japan). The aberration-corrected HAADF-STEM images and corresponding high-resolution EDS analyses were performed using a JEOL JEM-ARM300F atomic resolution electron microscope with a double spherical aberration corrector. Nitrogen adsorption/desorption isotherms were collected at 77 K on a Quadrasorb SI MP apparatus. Specific surface areas were calculated via the BET method. Diffuse reflectance spectra were recorded on a Cary 7000 (Agilent) spectrometer equipped with an integrating sphere attachment. The actual metal contents in the photocatalysts were determined by inductively coupled plasma-optical emission spectroscopy (ICP-OES, Varian 710).

C_2_H_6_-TPD profiles were obtained using an AutoChem II 2920 (Micromeritics Instrument Corporation). Photocatalysts (200.0 mg) were pretreated under a He atmosphere for 60 min at 150 °C, using a heating rate of 10 °C min^–1^. After cooling to 50 °C, photocatalysts were kept under a pure C_2_H_6_ atmosphere for 60 min to achieve adsorption saturation. Next, the samples were kept under a He flow (50 mL min^–1^) for 60 min to remove any weakly physically adsorbed ethane. Finally, the C_2_H_6_-TPD tests were carried out by heating the photocatalysts from 50 °C to 800 °C at a heating rate of 10 °C min^–1^ under a He carrier gas, with a mass spectrometer used for C_2_H_6_ detection. The test method for O_2_-TPD experiments was similar to C_2_H_6_-TPD tests, except that C_2_H_6_ is replaced by O_2_.

In situ EPR spectra were collected on a Bruker E500 spectrometer. Before the EPR tests, the catalyst was pre-treated at 150 °C for 2 h under an Ar flow to remove any surface adsorbed species. 50.0 mg of photocatalyst was loaded into a quartz tube and evacuated. Then, Ar or C_2_H_6_ gas was introduced into the EPR tube. EPR spectra were recorded in the dark and light conditions at 110 K.

Time-resolved online MS experiments were performed in a homemade fixed-bed stainless steel flow reactor (volume = 56.0 mL) with a quartz window on the top for irradiation of photocatalysts. The reactor was coupled to a mass spectrometer (SPIMS 2000, Hexin mass spectrum). Before each test, 20.0 mg of photocatalyst was uniformly dispersed on a glass fiber membrane and the modified membrane was then pretreated in an Ar gas flow at 200 °C for 2 h to remove any impurities from the membrane.

In situ Fourier transform infrared spectroscopy (FT-IR) data were collected on a Bruker Vertex 70 V FT-IR spectrometer equipped with a narrowband HgCdTe detector and a transmission reaction chamber (Harrick) connected to an evacuation line (∼ 10^–7^ mbar). 5.0 mg of PdZn-ZnO photocatalyst was pressed into a self-supported pellet (7.0 mm in diameter) and placed in the transmission chamber. In a typical in situ FT-IR experiment, the photocatalyst pellet was first purged with Ar (100 mL min^–1^) for 30 min to remove impurities adsorbed on the surface. Then, reactant gas (3 vol.% C_2_H_6_, 0.4 vol.% O_2_, balanced with Ar, 30 mL min^–1^) was introduced into the chamber. After 30 min of reactant adsorption, a background FT-IR spectrum was then recorded. Further FT-IR spectra were then collected every minute in the reaction atmosphere under illumination from a 150 mW continuous diode laser (375 nm), which served as the light source. Each spectrum was recorded by averaging 200 scans collected at a scanning velocity of 40 kHz and a resolution of 4 cm^–1^.

### Photocatalytic activity measurements

Photocatalytic ODHE tests were conducted in a custom-built fixed-bed stainless steel flow reactor (volume = 56.0 mL) with a quartz window on the top for light irradiation (Supplementary Fig. 44). Typically, photocatalyst (5.0 mg) was uniformly spread on a glass fiber membrane (Whatman, GE Healthcare Life Sciences, catalog number 1823-047) and then placed in the reactor (parallel to the quartz window). Then, a reaction gas comprising C_2_H_6_ (5 vol.% in Ar, 18 mL min^–1^) and O_2_ (1 vol.% in Ar, 12 mL min^–1^) with a total flow rate of 30 mL min^–1^ was introduced to the reactor. After purging for 30 min to remove the air in the reactor. a 365 nm LED lamp (100 W 365 nm LED, Beijing PerfectLight Technology Co., Ltd., PLS-LED100C) was applied as light source to drive the reaction. Gas samples in the outlet were analyzed using a gas chromatograph (Shimadzu GC-2014, Shimadzu Co., Japan) equipped with three channels. The first channel analyzed hydrocarbons in an HP PLOT Al_2_O_3_ column with He as a carrier gas and a flame ionization detector (FID). The second channel analyzed CO_2_, N_2_, Ar, O_2_, CH_4_, and CO with a combination of micropacket Haysep Q, H-N, and Molsieve 13× columns using He as the carrier gas and a thermal conductivity detector (TCD). The third channel analyzes H_2_ using a micropacket HayeSep Q and Molsieve 5 Å column with N_2_ as a carrier gas and a TCD detector. For the batch-reactor test, a batch type reactor (volume = 56.0 mL) was used. After evacuation of the reaction system, reaction gas (3 vol.% C_2_H_6_, 0.4 vol.% O_2_, balanced with Ar) was introduced into the reactor until the pressure reached 0.3 MPa, after which the photocatalyst was exposed to the 365 nm LED light source.

The C_2_H_4_ selectivity is calculated according to the following equation.

1 $${{{{{{\rm{C}}}}}}}_{2}{{{{{{\rm{H}}}}}}}_{4}\,{{{{{\rm{selectivity}}}}}}\, \left(\%\right)=\frac {n({{{{{{\rm{C}}}}}}}_{2}{{{{{{\rm{H}}}}}}}_{4})}{n({{{{{{\rm{C}}}}}}}_{2}{{{{{{\rm{H}}}}}}}_{4})+\frac{1}{2} \times \left[n({{{{{\rm{C}}}}}}{{{{{{\rm{H}}}}}}}_{4})+n({{{{{{\rm{CO}}}}}}}_{2})\right]} \times 100\%$$

where $n({{{{{{\rm{C}}}}}}}_{2}{{{{{{\rm{H}}}}}}}_{4})$, $n({{{{{\rm{C}}}}}}{{{{{{\rm{H}}}}}}}_{4})$ and $n({{{{{\rm{CO}}}}}}_{2})$ represent the moles of C_2_H_4_, CH_4_ and CO_2_ at the outlet, respectively. The conversion rate is calculated based on products according to the following equation.

2 $${{{{{{\rm{C}}}}}}}_{2}{{{{{{\rm{H}}}}}}}_{6}\,{{{{{\rm{conversion}}}}}}\, \left(\%\right)=\frac{n({{{{{{\rm{C}}}}}}}_{2}{{{{{{\rm{H}}}}}}}_{4})+\frac{1}{2}\times \left[n({{{{{\rm{C}}}}}}{{{{{{\rm{H}}}}}}}_{4})+n({{{{{{\rm{CO}}}}}}}_{2})\right]}{{n}_{0}({{{{{{\rm{C}}}}}}}_{2}{{{{{{\rm{H}}}}}}}_{6})}\times 100\%$$

where ${n}_{0}({{{{{{\rm{C}}}}}}}_{2}{{{{{{\rm{H}}}}}}}_{6})$ represents the moles of C_2_H_6_ at the inlet. The carbon balance is calculated according to the following equation.

3 $${{{{{\rm{Carbon}}}}}}\,{{{{{\rm{balance}}}}}}\, \left(\%\right)=\frac{n({{{{{{\rm{C}}}}}}}_{2}{{{{{{\rm{H}}}}}}}_{4})+\frac{1}{2}\times \left[n\left({{{{{\rm{C}}}}}}{{{{{{\rm{H}}}}}}}_{4}\right)+n({{{{{{\rm{CO}}}}}}}_{2})\right]+{n}_{1}({{{{{{\rm{C}}}}}}}_{2}{{{{{{\rm{H}}}}}}}_{6})}{{n}_{0}({{{{{{\rm{C}}}}}}}_{2}{{{{{{\rm{H}}}}}}}_{6})}\times 100\%$$

where ${n}_{1}\left({{{{{{\rm{C}}}}}}}_{2}{{{{{{\rm{H}}}}}}}_{6}\right)$ represent the moles of C_2_H_6_ at the outlet, respectively. The AQE was calculated according to the following equation.

4 $${{{{{\rm{AQE}}}}}}=\frac{n({{{{{\rm{electrons}}}}}})}{n({{{{{\rm{photons}}}}}})}\times 100\%$$

where *n*(electrons) and *n*(photons) represent the number of reacted electrons and the number of incident photons, respectively. According to the chemical equation (2C_2_H_6_ + O_2_ → 2C_2_H_4_ + 2H_2_O, 2C_2_H_6_ + 7O_2_ →  4CO_2_ + 6H_2_O), *n*(electrons) = 2*n*(C_2_H_4_) + 7*n*(CO_2_), where *n*(C_2_H_4_) and *n*(CO_2_), represent the moles of produced C_2_H_4_ and CO_2_, respectively. *n*(photons) *= IAt/E*, where *I*, *A*, t, and *E* represent light intensity (W cm^–2^), irradiation area (12.57 cm^–2^), irradiation time (s) and photon energy (J), respectively. *E* = *hc/λ*, where *h*, *c*, and *λ* represent Planck’s constant, light speed, and monochromatic light wavelength, respectively. The light intensity *I* at different wavelengths (*λ* = 350, 365, 380, 400, 450, 500, 600, and 700 nm) was measured to be 51.3, 52.6, 50.2, 49.8, 50.4, 50.5, 51.3, and 49.8 mW cm^–2^ by xenon lamp (300 W Xe lamp, Beijing Perfectlight Technology Co. Ltd, PLS-SXE300D) source and band pass filter (Supplementary Fig. 45). Since the surface temperature of the catalyst was around room temperature under monochromatic light irradiation, the contribution of heat induced by illumination during AQE tests was not considered.

### Computational details

First-principles spin-polarized calculations were performed using the Vienna ab initio Simulation Program (VASP)^47,48^. The generalized gradient approximation (GGA) in the Perdew-Burke-Ernzerhof (PBE) form and a cutoff energy of 700 eV for planewave basis set were adopted^49^. A 3 × 3 × 1 Monkhorst-Pack^50^ k grid was used for sampling the Brillouin zones in the structure calculations. Ion-electron interactions were described by the projector augmented wave (PAW) method^51^. The convergence criteria for structure optimization was a maximum force less than 0.02 eV Å^–1^ on each atom with an energy change less than 1 × 10^–5^ eV. The DFT-D3 semiempirical correction was described via Grimme’s scheme method^52,53^.

Since the main exposed facets of ZnO were (101), a ZnO(101) model was used for the calculations. The ZnO(101) surface was modelled by a 3 × 3 unit cell based on an initial structure with lattice parameters a = 18.4 Å, b = 19.5 Å, c = 25.4 Å, α = β = 90°, γ = 105°. Pd_15_Zn_15_ cluster with fifteen Pd atoms and fifteen Zn atom was used to simulate a PdZn intermetallic nanoparticle, whilst Pd_30_ cluster with thirty Pd atoms was used to simulate a Pd nanoparticle. The above Pd_15_Zn_15_ and Pd_30_ clusters were placed on ZnO(101) surface to form models Pd_15_Zn_15_-ZnO(101) and Pd_30_-ZnO(101), respectively. Models similar to Pd_15_Zn_15_-ZnO(101) were used to build Pd_15_Cu_15_-ZnO(101), Pd_15_Ni_15_-ZnO(101), and Pd_15_In_15_-ZnO(101).

To calculate the kinetic energy barrier of chemical reactions, the climbing image nudged elastic band (CI-NEB) method was used to search for the TS^54,55^, with convergence criteria a force below 0.05 eV Å^−1^. *E*_a_ was calculated using *E*_a_ = *E*_TS_ *– E*_IS_, where *E*_TS_ and *E*_IS_ represents the energy of TS and the initial state.

### Supplementary information

Supplementary Information

Peer Review File

### Source data

Source Data

## Data Availability

The datasets generated and/or analyzed during the current study are available from the corresponding author on reasonable request. Received: ((will be filled in by the editorial staff)) Accepted: ((will be filled in by the editorial staff)) Published online: ((will be filled in by the editorial staff)) Source data are provided with this paper.
