# Supplementary material for: Photocatalytic ethylene production by oxidative dehydrogenation of ethane with dioxygen on ZnO-supported PdZn intermetallic nanoparticles
Source: Nat Commun. 2024 Jan 26;15:789. doi: 10.1038/s41467-024-45031-6 (PMC10817976; doi:10.1038/s41467-024-45031-6)
Supplement: Supplementary file 1 — Supplementary Information [file 41467_2024_45031_MOESM1_ESM.pdf]

Supplementary Information

Photocatalytic ethylene production by oxidative dehydrogenation of ethane with dioxygen on ZnO-supported PdZn intermetallic nanoparticles

Pu Wang[1,2]*, Xingyu Zhang*[2,3]*, Run Shi*[1]**, Jiaqi Zhao*[1,2]*, Geoffrey I.N. Waterhouse*[4]*, Junwang Tang*[5,6]*, Tierui Zhang*[1,2]***

[1]Key Laboratory of Photochemical Conversion and Optoelectronic Materials, Technical Institute of Physics and Chemistry, Chinese Academy of Sciences, Beijing 100190, China.

[2]Center of Materials Science and Optoelectronics Engineering, University of Chinese Academy of Sciences, Beijing 100049, China.

[3]Functional Crystals Lab, Technical Institute of Physics and Chemistry, Chinese Academy of Sciences, Beijing 100190, China.

[4]School of Chemical Sciences, The University of Auckland, Auckland 1142, New Zealand.

[5]Department of Chemical Engineering, University College London, London WC1E 7JE, U.K.

[6]Industrial Catalysis Center, Department of Chemical Engineering, Tsinghua University, Beijing 100084, China.

Keywords: photocatalysis, oxidative dehydrogenation of ethane, ethylene, PdZn intermetallic nanoparticles

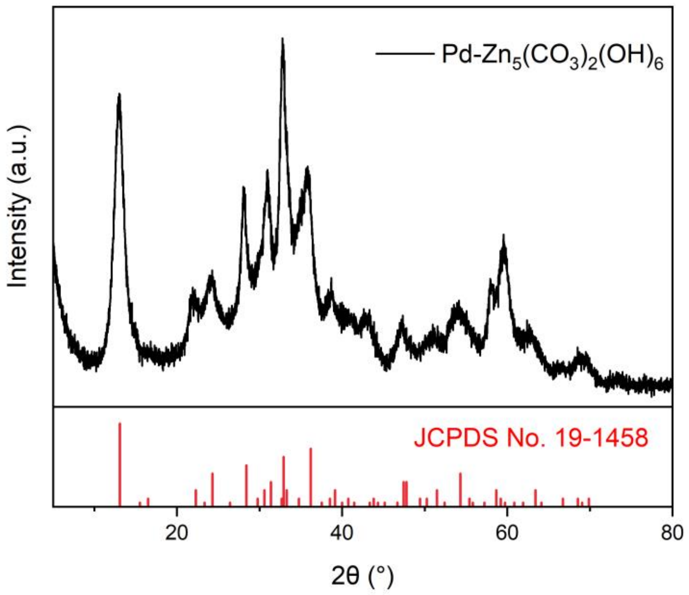

Supplementary Figure 1. XRD pattern for Pd-doped $Zn_5(CO_3)_2(OH)_6$.

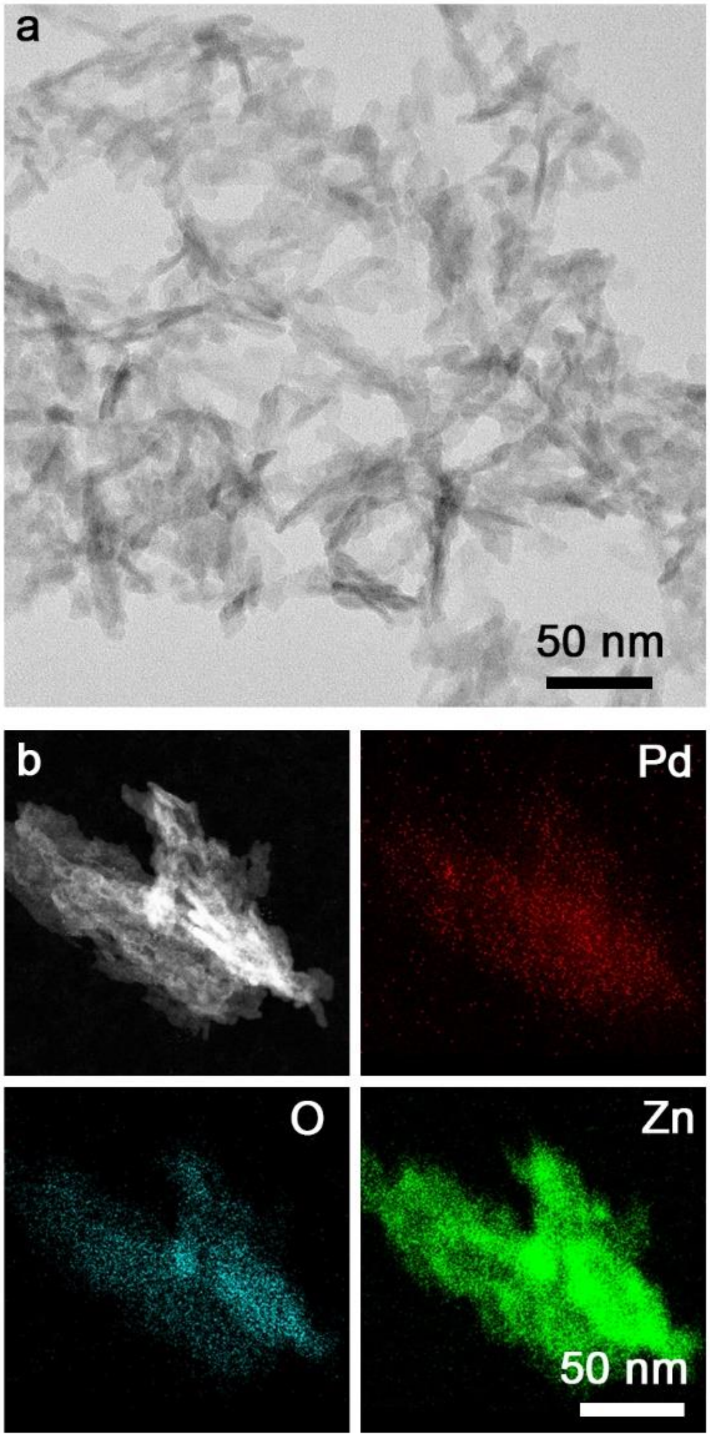

Supplementary Figure 2. Characterization for Pd-$Zn_5(CO_3)_2(OH)_6$. (a) TEM image and (b) EDS elemental maps for Pd-doped $Zn_5(CO_3)_2(OH)_6$.

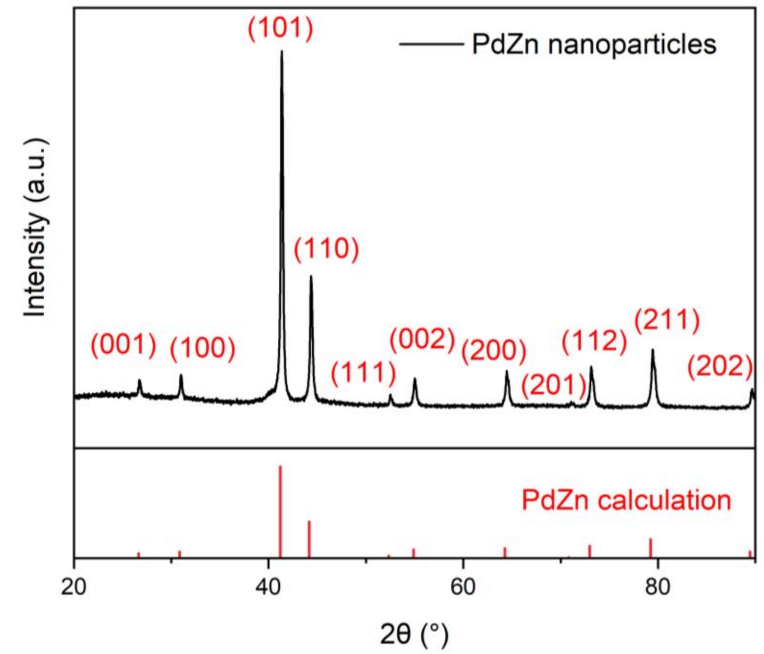

Supplementary Figure 3. XRD pattern of the PdZn nanoparticles obtained by dissolution of the ZnO support in PdZn-ZnO. The calculated PdZn XRD pattern was obtained using VESTA software, based on the crystal structure shown in Fig. 1a.

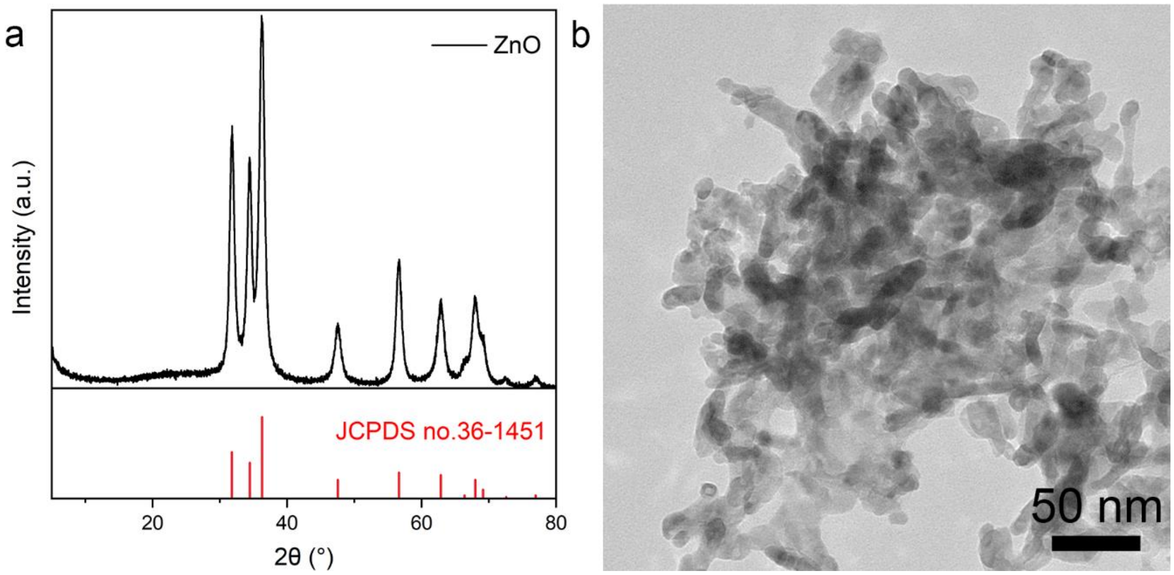

Supplementary Figure 4. Characterization for ZnO. (a) XRD pattern and (b) TEM image for ZnO.

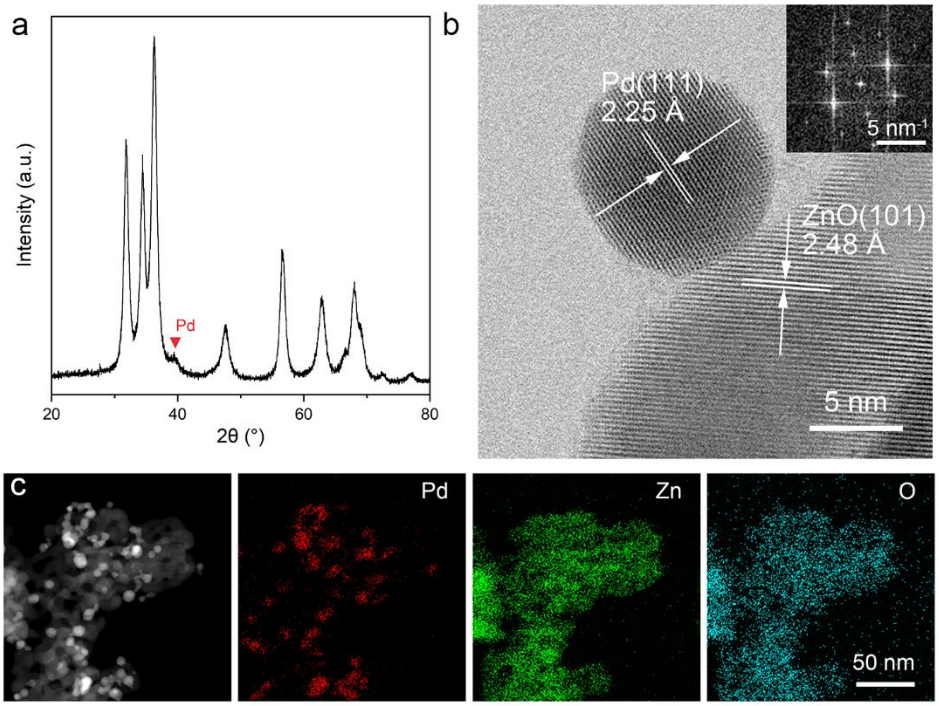

Supplementary Figure 5. Characterization for Pd-ZnO. (a) XRD pattern, (b) high-resolution TEM image, and (c) EDS elemental maps for Pd-ZnO. The inset in (b) shows the fast Fourier transform pattern for a Pd nanoparticle.

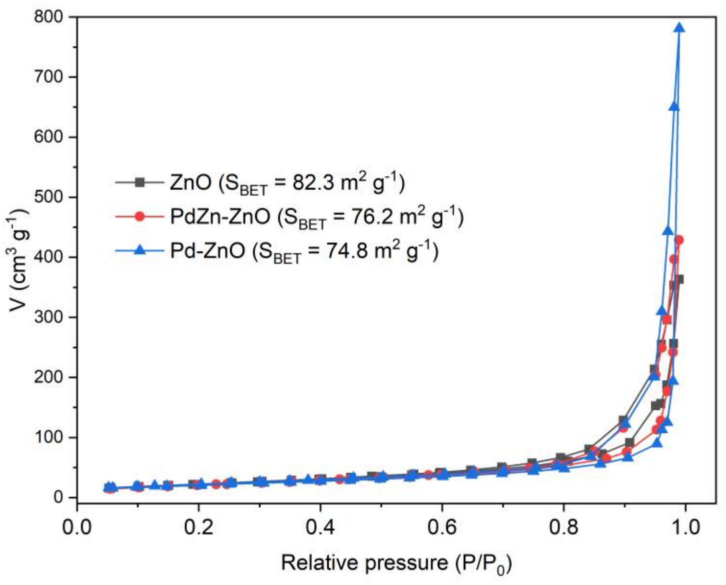

Supplementary Figure 6. N_2 adsorption/desorption isotherms for ZnO, Pd-ZnO, and PdZn-ZnO. S_{BET} refers to Brunauer-Emmett-Teller (BET) specific surface area.

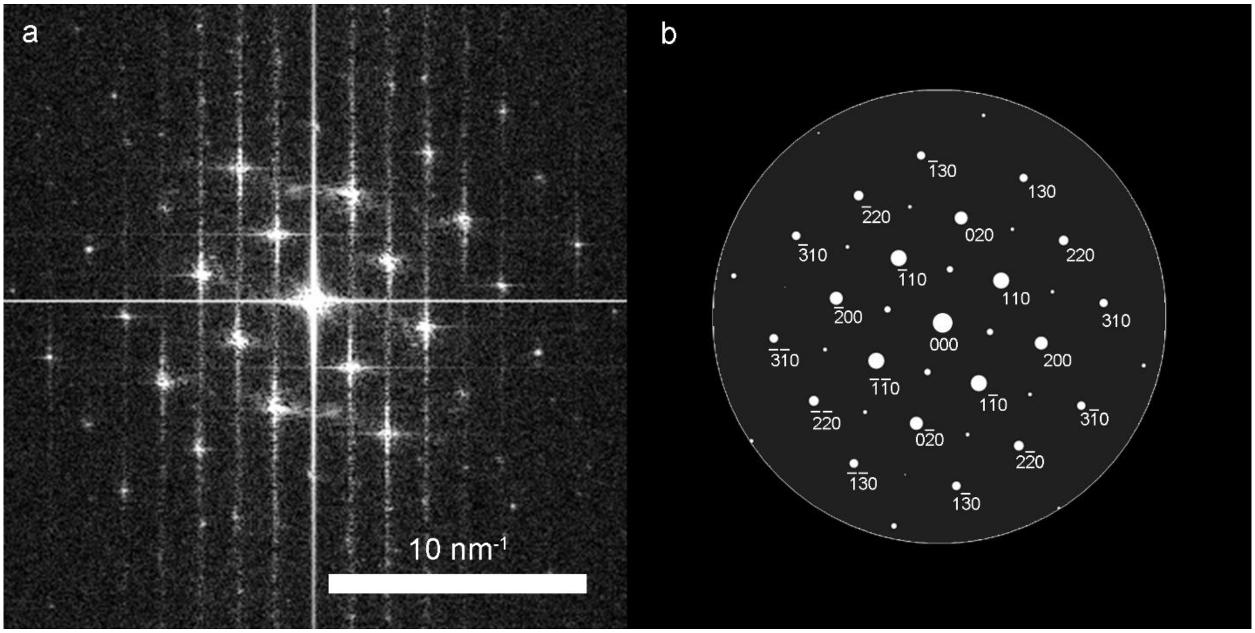

Supplementary Figure 7. Actual and simulated Fast-Fourier Transform pattern. (a) Fast-Fourier Transform pattern of the aberration-corrected HAADF-STEM image of a PdZn nanoparticle. (b) Fast-Fourier Transform image simulation results for a PdZn intermetallic nanoparticle.

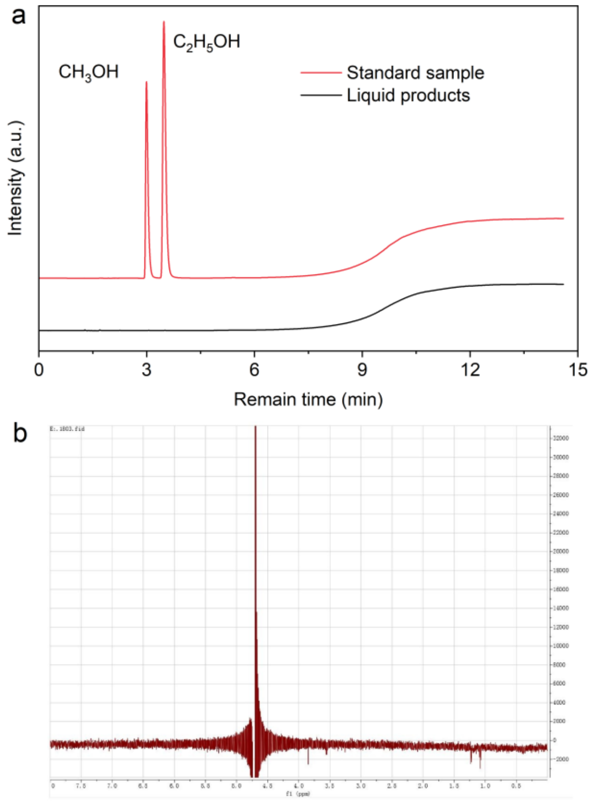

Supplementary Figure 8. Liquid products for photocatalytic ODHE on PdZn-ZnO. (a) Gas chromatography spectrum and (b) ^{1}H nuclear magnetic resonance spectroscopy (NMR) spectrum of possible carbon-containing liquid products.

A glass beaker containing 20 mL of deionized water was connected to the outlet of the photoreactor and used to collect the liquid products. After 4 h continuous reaction, the water in the glass beaker was used to clean the photoreactor walls and catalyst membrane to ensure collection of all liquid products. Subsequently, the water was subjected to gas chromatograph and ^{1}H NMR analysis.

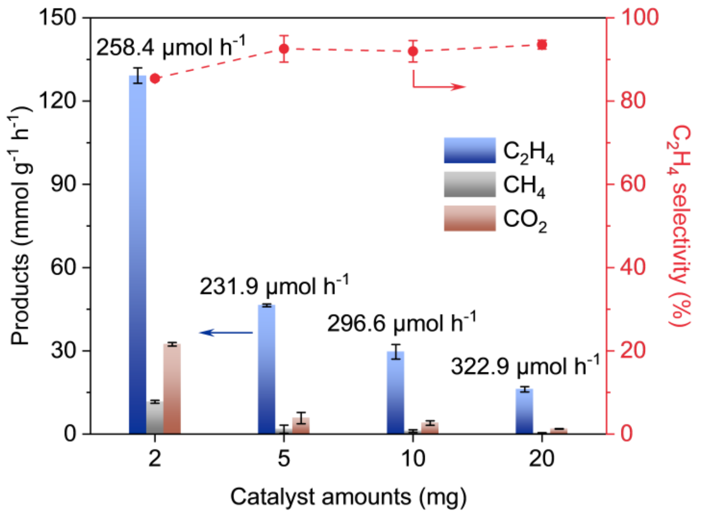

Supplementary Figure 9. Photocatalytic ODHE performance of PdZn-ZnO as a function of the photocatalyst dosage.

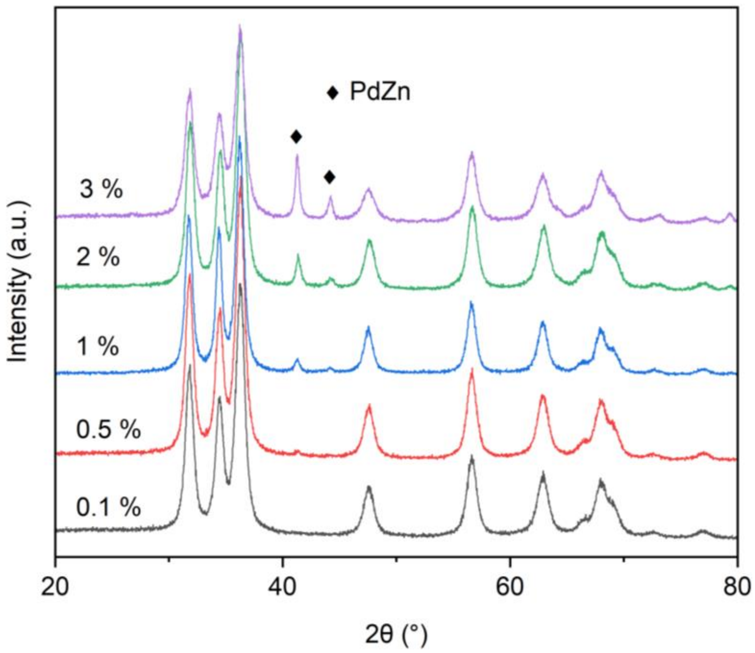

Supplementary Figure 10. XRD patterns for PdZn-ZnO catalysts with different Pd loadings (in wt.%).

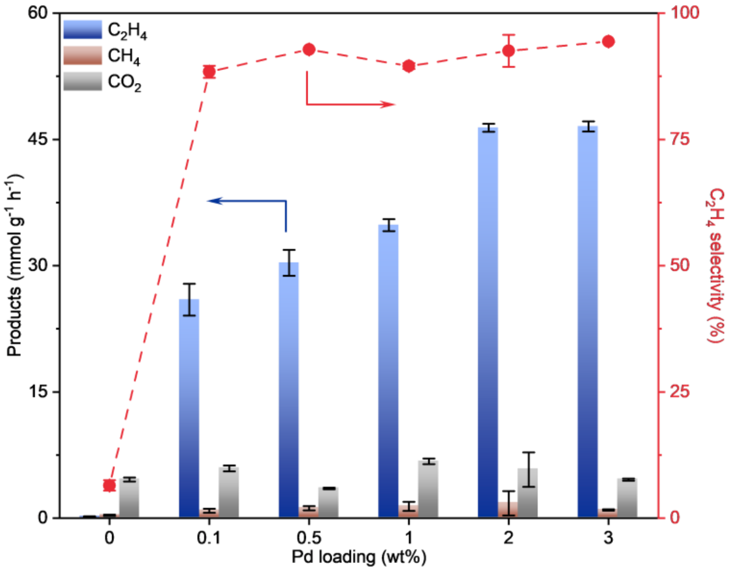

Supplementary Figure 11. Photocatalytic ODHE performance over PdZn-ZnO catalysts with different Pd loadings.

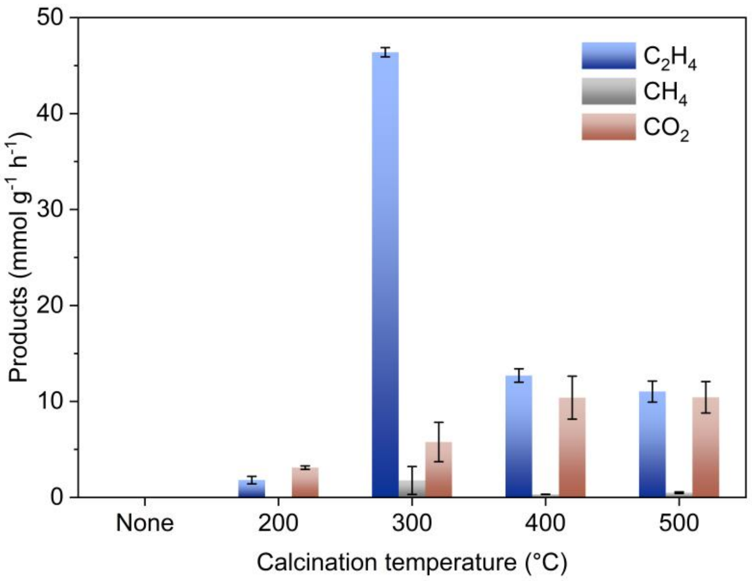

Supplementary Figure 12. Photocatalytic ODHE performance with Pd-$Zn_5(CO_3)_2(OH)_6$ calcinated at different temperatures.

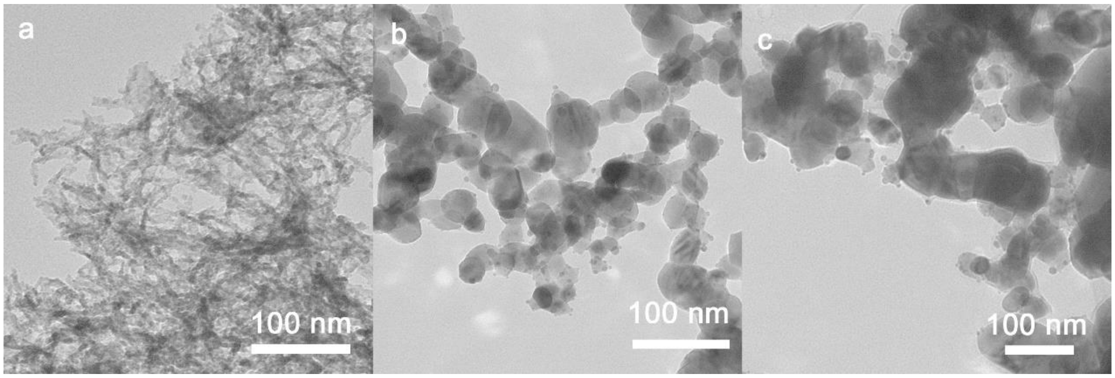

Supplementary Figure 13. TEM images for Pd-$Zn_5(CO_3)_2(OH)_6$ calcinated at different temperatures. (a) 200 °C, (b) 400 °C, (c) 500 °C. The samples calcined at 200 °C still retained the nanosheet morphology of the precursor, while those calcined at 400 °C and 500 °C exhibited noticeable agglomeration and sintering of ZnO nanoparticles.

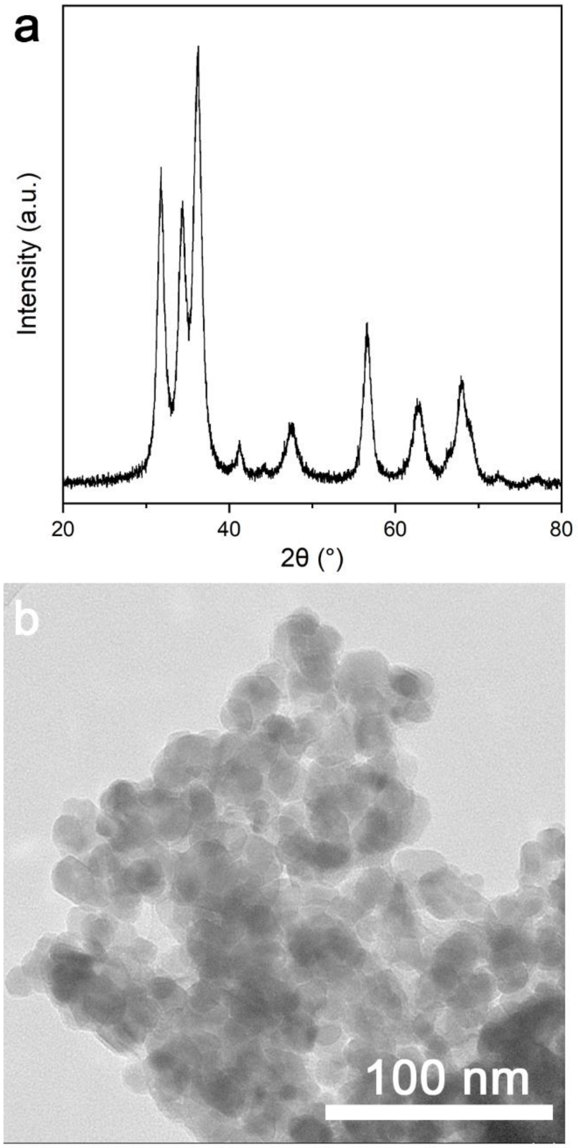

Supplementary Figure 14. Characterization for PdZn-ZnO-mix. (a) XRD pattern and (b) TEM image for PdZn-ZnO-mix.

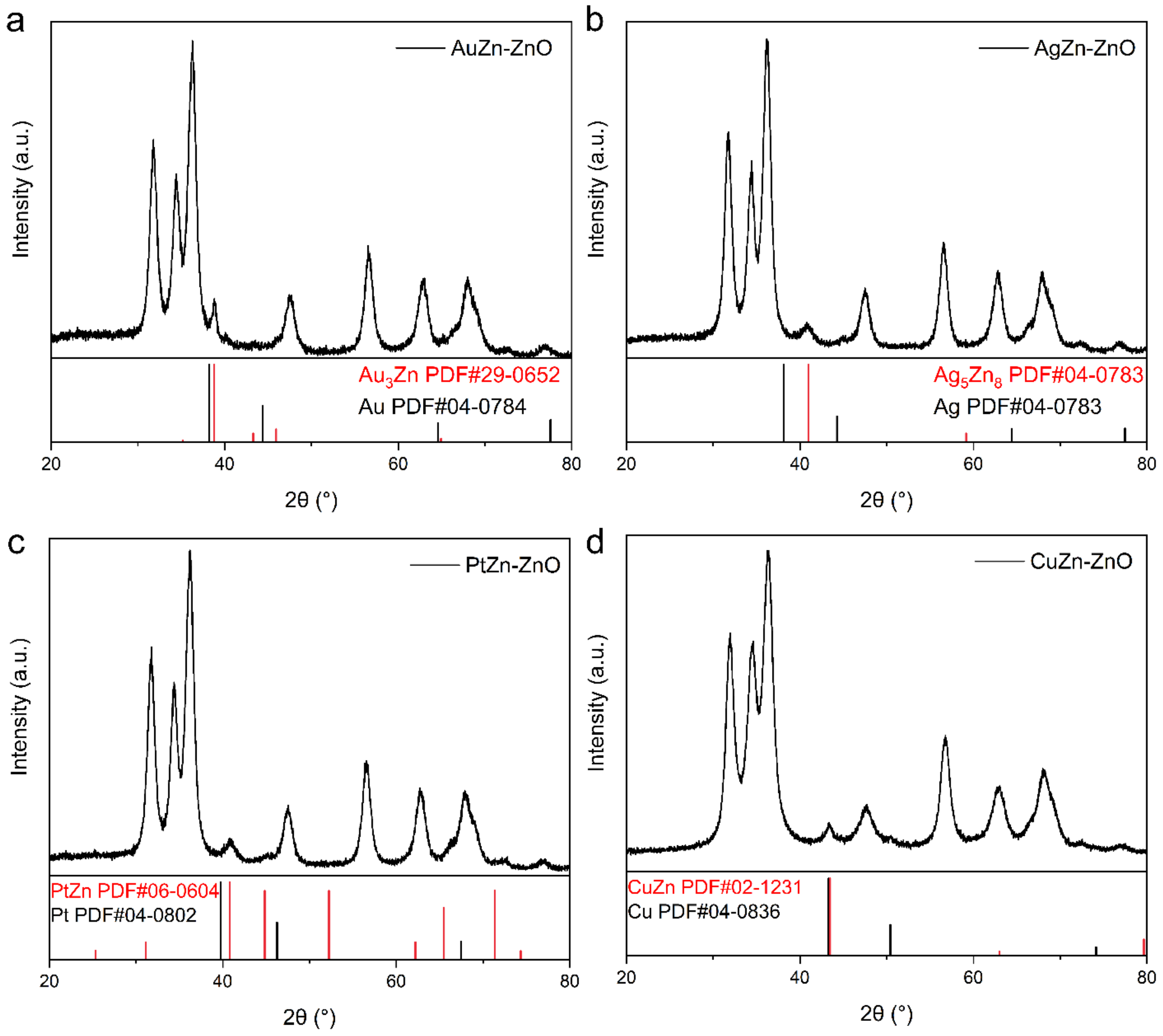

Supplementary Figure 15. XRD patterns for MZn-ZnO (M = Ag, Au, Pt, and Cu). (a) AuZn-ZnO, (b) AgZn-ZnO, (c) PtZn-ZnO, (d) CuZn-ZnO.

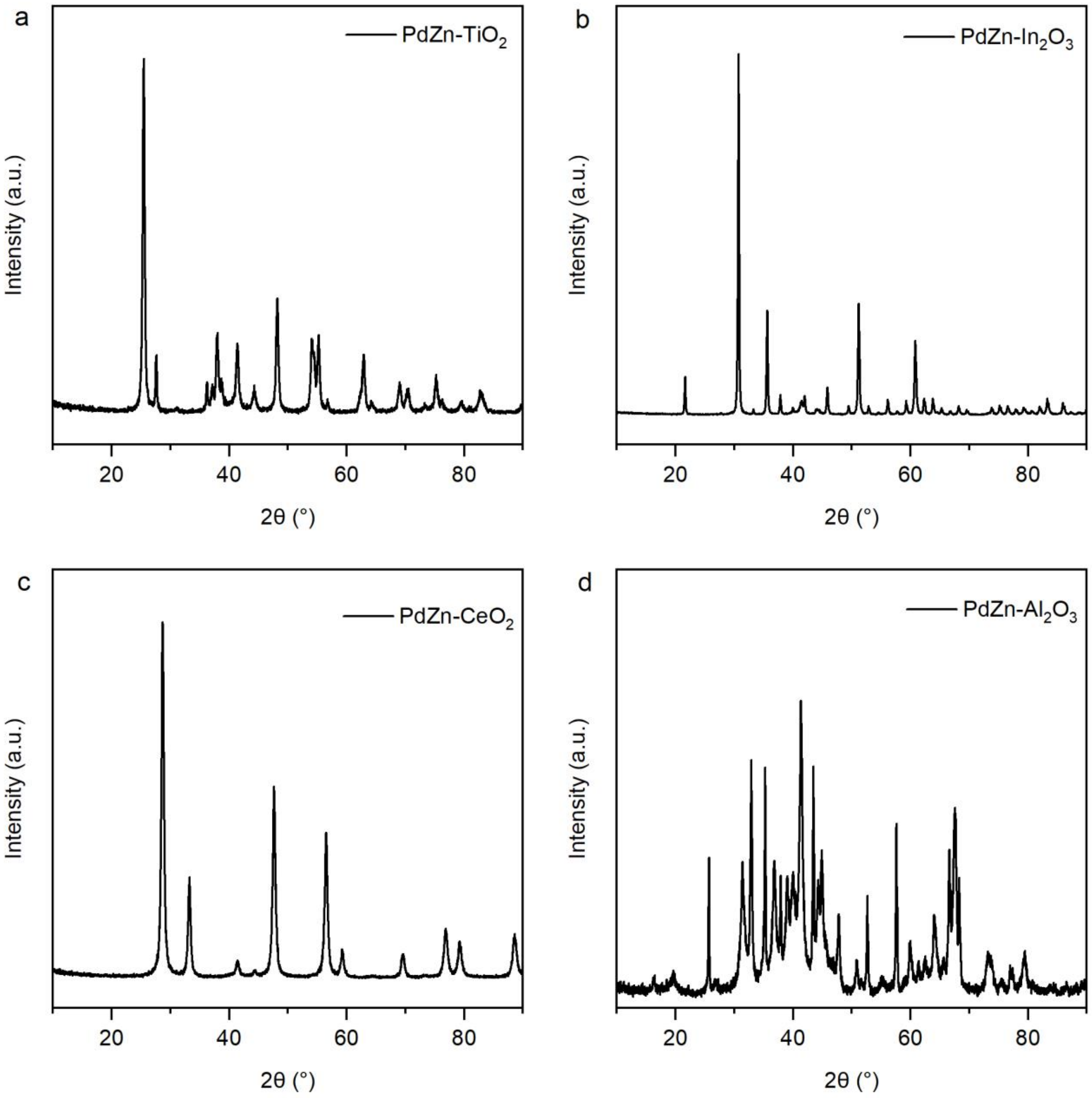

Supplementary Figure 16. XRD patterns for PdZn nanoparticles supported on different metal oxides. (a) PdZn-TiO_2, (b) PdZn-In_2O_3, (c) PdZn-CeO_2, (d) PdZn- Al_2O_3.

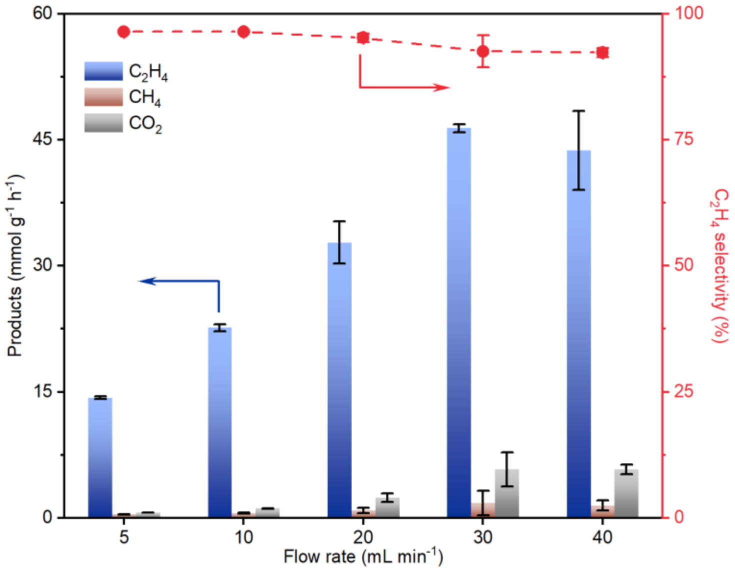

Supplementary Figure 17. Photocatalytic ODHE performance over PdZn-ZnO at different total flow rates. The C_2H_6 (5 vol.% in Ar)/O_2 (1 vol.% in Ar) feed gas ratio was fixed at 18/12.

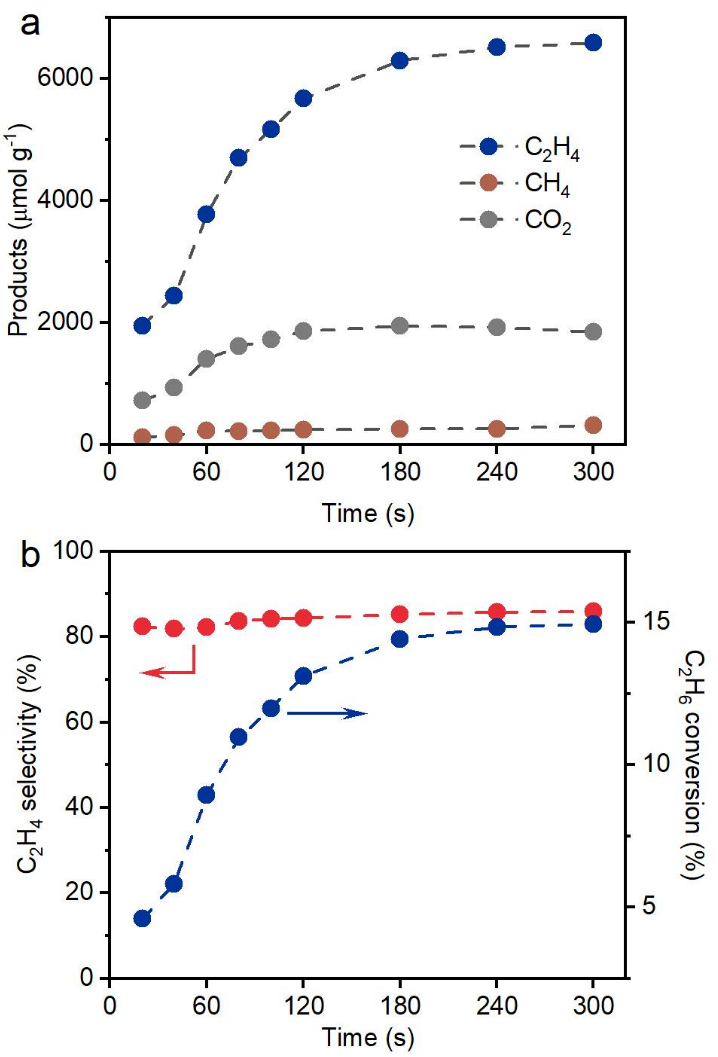

Supplementary Figure 18. Photocatalytic ODHE activity over PdZn-ZnO in a batch reactor. (a) The amount of C_2H_4, CH_4, and CO_2 production over time. (b) C_2H_4 selectivity and C_2H_6 conversion over time. Reaction condition: 5.0 mg photocatalyst, 0.3 MPa, 3.0 vol.% C_2H_6 + 0.4 vol.% O_2 (balanced with Ar), 365 nm LED, 600 mW cm^{-2}.

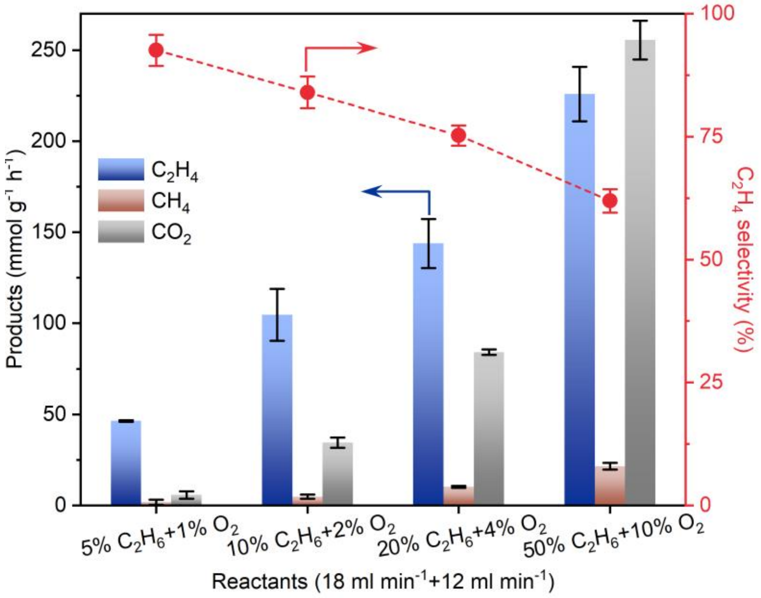

Supplementary Figure 19. Photocatalytic ODHE performance at varied reactant concentrations.

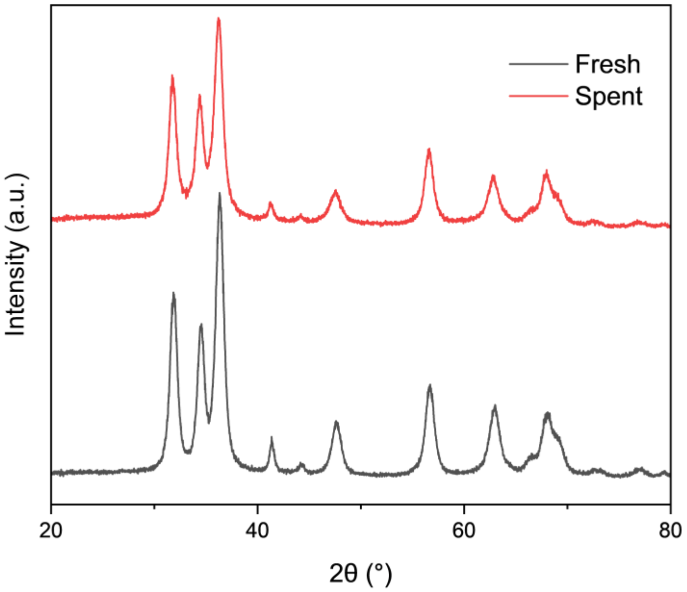

Supplementary Figure 20. XRD patterns of fresh and spent PdZn-ZnO.

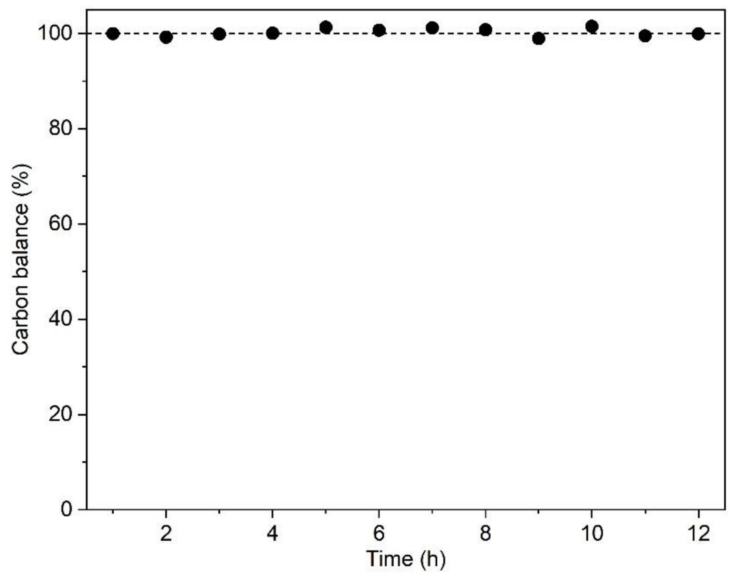

Supplementary Figure 21. Carbon balance for the stability test in Fig. 2c.

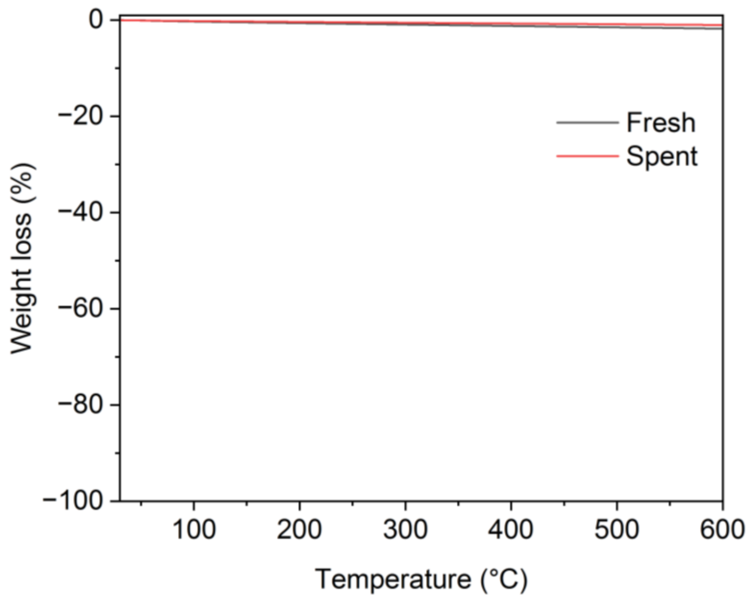

Supplementary Figure 22. Thermogravimetric analysis curves of fresh and spent PdZn-ZnO. Samples were heated at 10 °C min^{-1} in O_2.

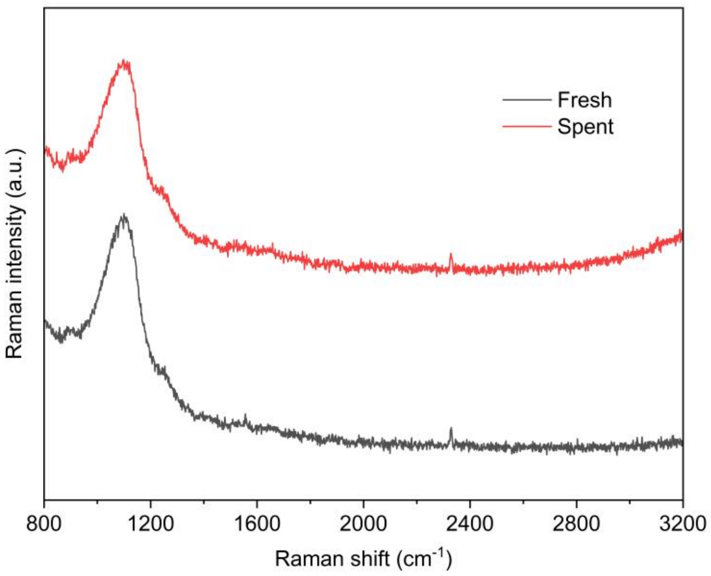

Supplementary Figure 23. Raman spectra of fresh and spent PdZn-ZnO.

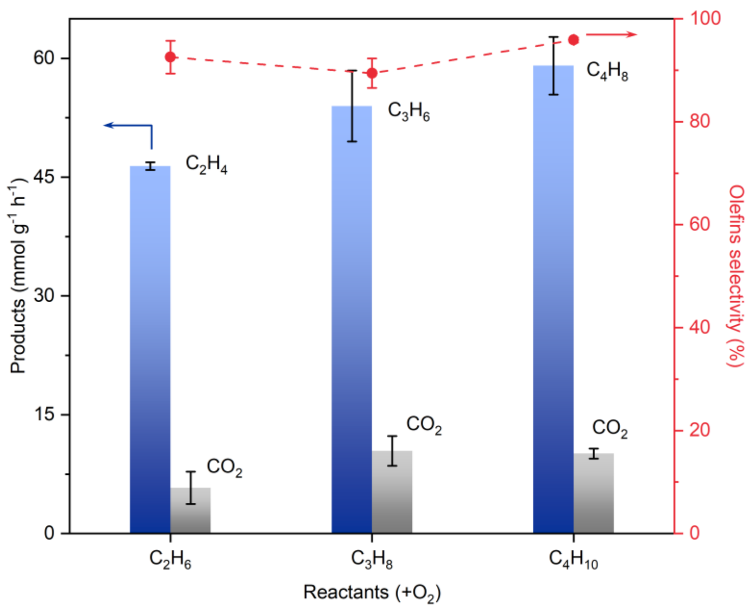

Supplementary Figure 24. Photocatalytic oxidative dehydrogenation of light alkanes over PdZn-ZnO. Reaction condition: 5.0 mg PdZn-ZnO, alkane (5 vol.% in Ar, 18 mL min^{-1}) + O_2 (1 vol.% in Ar, 12 mL min^{-1}), 365 nm LED, 600 mW cm^{-2}.

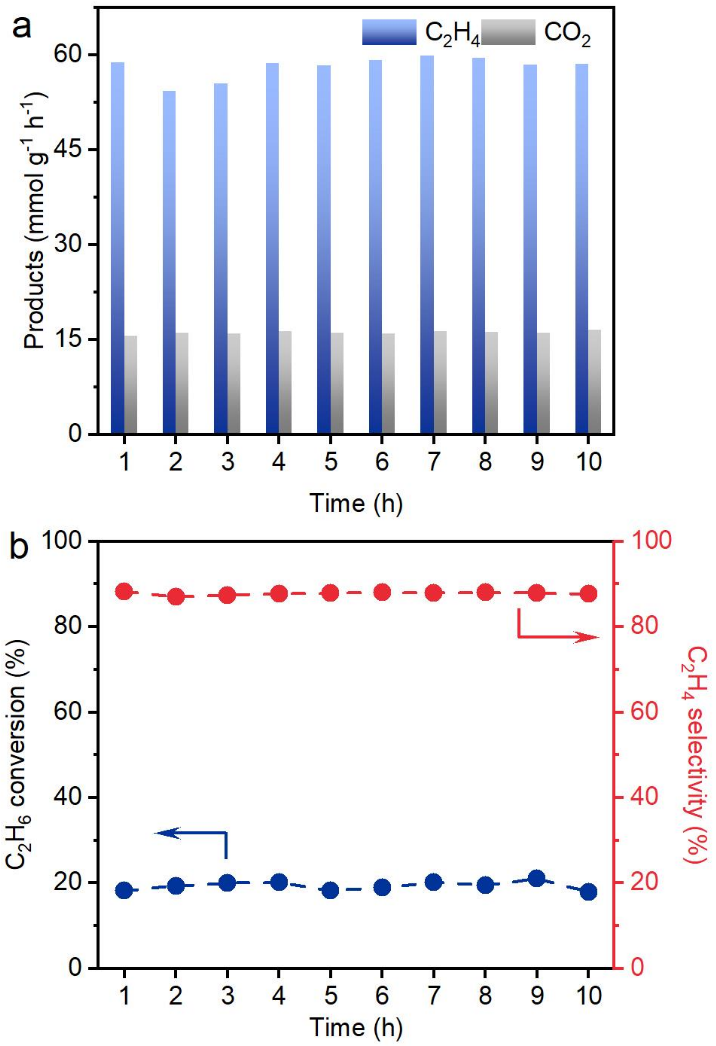

Supplementary Figure 25. Photocatalytic ODHE performance over PdZn-ZnO using simulated shale gas. (a) Production rate for C_2H_4 and CO_2. (b) C_2H_6 conversion and C_2H_4 selectivity. Reaction condition: 5.0 mg PdZn-ZnO, simulated shale gas (5 vol.% C_2H_6, 45 vol.% CH_4, in Ar, 18 mL min^{-1}) + O_2 (1 vol.% in Ar, 12 mL min^{-1}), 365 nm LED, 600 mW cm^{-2}.

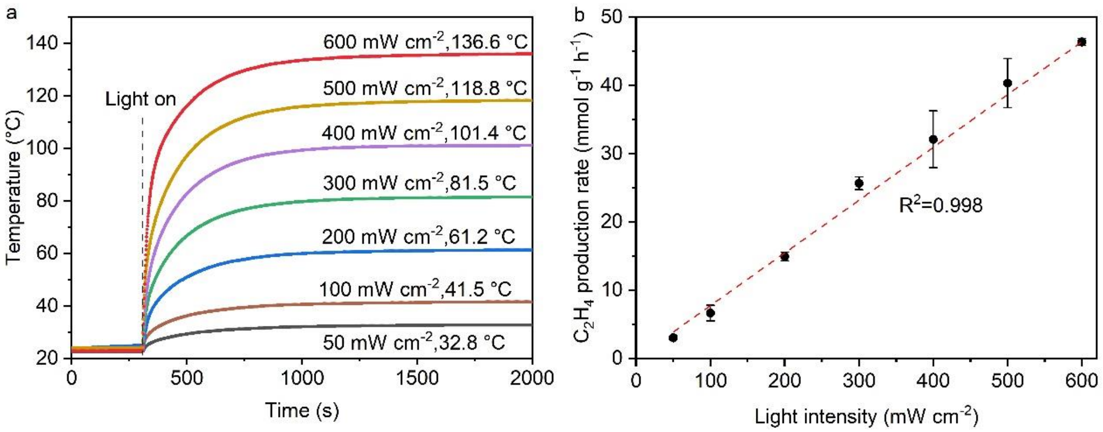

Supplementary Figure 26. Surface temperatures and C_2H_4 production rate under different light intensities. (a) Surface temperatures of PdZn-ZnO measured by a thermometer during photocatalytic ODHE under different light intensities. (b) C_2H_4 production rate under different light intensities.

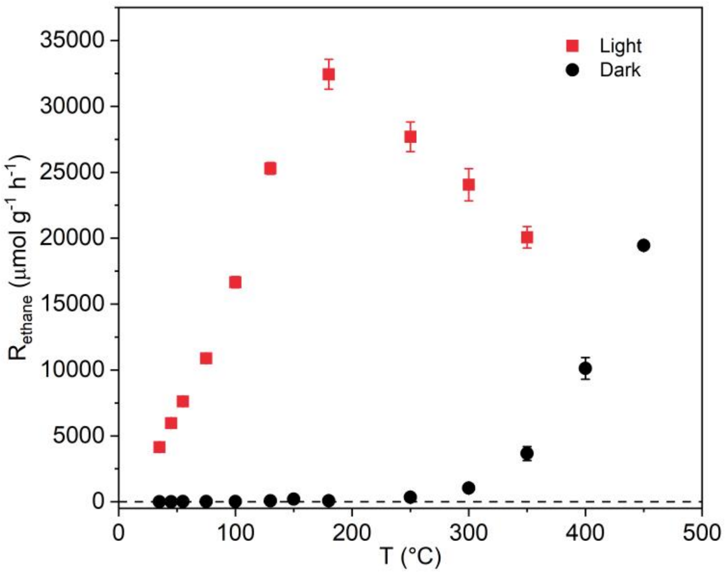

Supplementary Figure 27. C_2H_6 reaction rate under different temperatures. Temperature dependence of C_2H_6 reaction rate over PdZn-ZnO in the dark and light conditions. Reaction condition: 5.0 mg photocatalyst, C_2H_6 (5 vol.% in Ar, 18 mL min^{-1}) + O_2 (1 vol.% in Ar, 12 mL min^{-1}), 365 nm LED, 55.9 mW cm^{-2}.

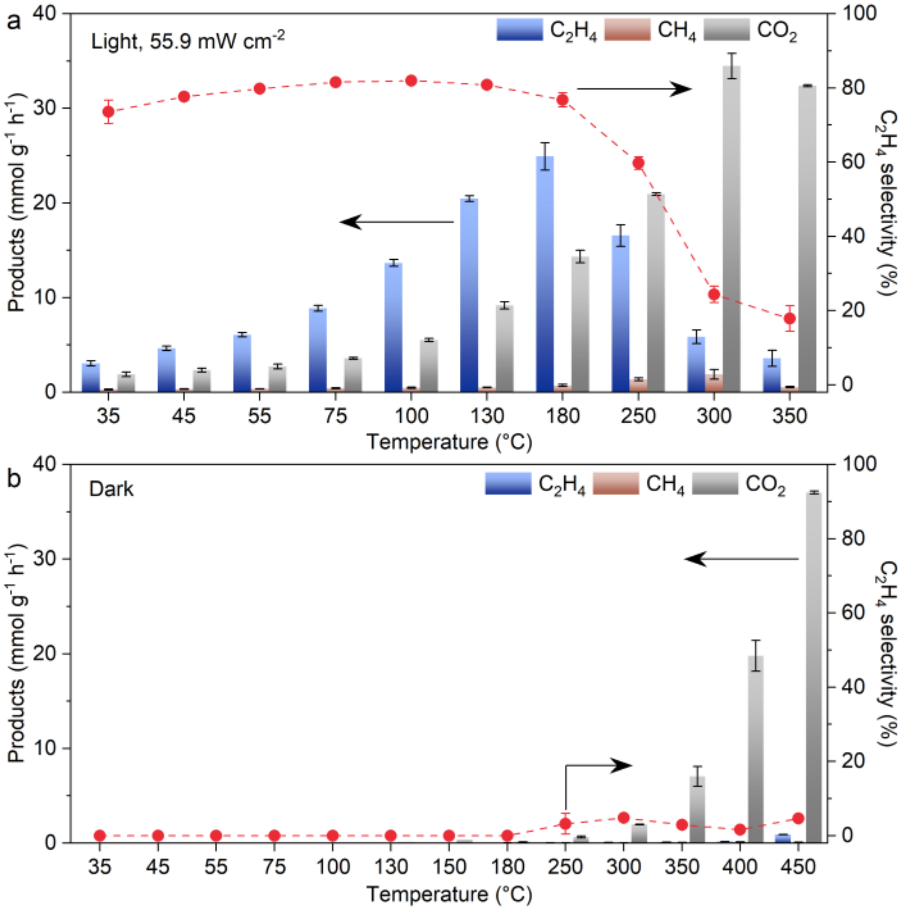

Supplementary Figure 28. ODHE performance under different temperatures with or without light irradiation. (a) Temperature-dependent photocatalytic ODHE performance. Light intensity = 55.9 mW cm^{-2}. (b) Temperature-dependent ODHE performance in the absence of light.

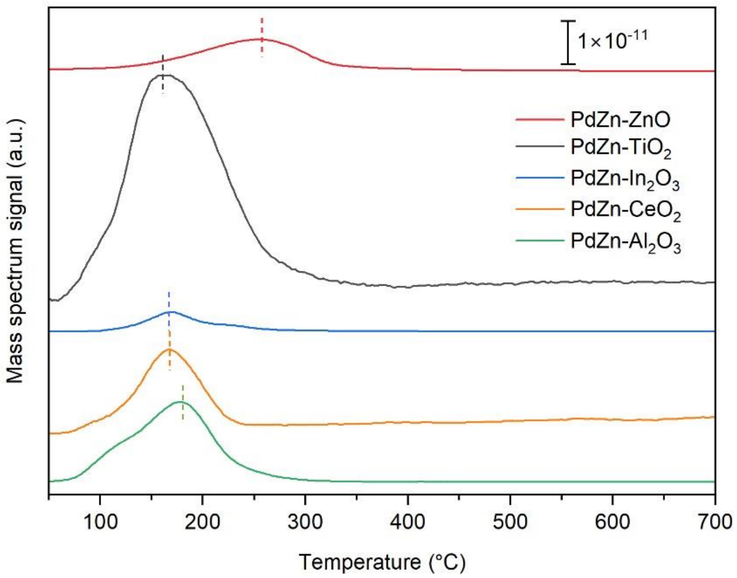

Supplementary Figure 29. Investigation on ethane adsorption behavior. C_2H_6-TPD spectra of PdZn-ZnO, PdZn-TiO_2, PdZn-In_2O_3, PdZn-CeO_2, and PdZn-Al_2O_3.

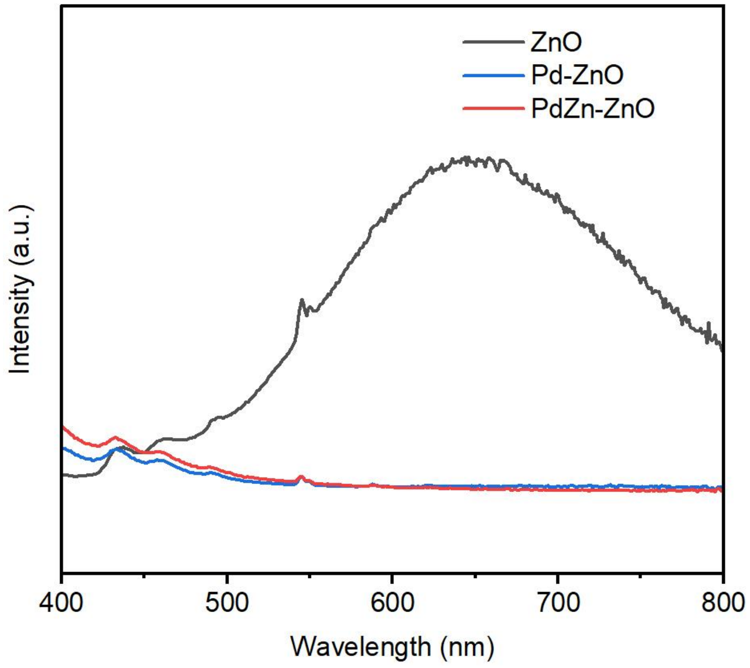

Supplementary Figure 30. PL spectra of ZnO, Pd-ZnO, and PdZn-ZnO under 365 nm excitation.

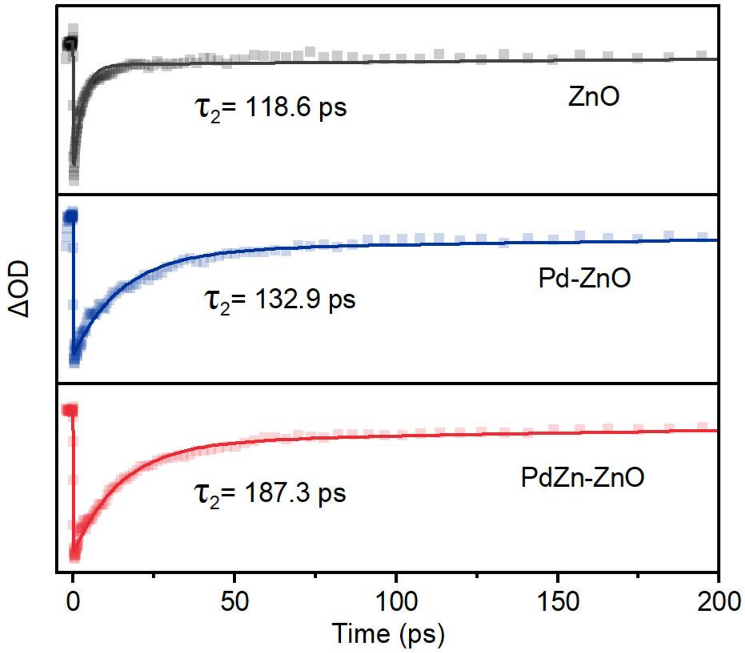

Supplementary Figure 31. Time-resolved TA spectra as a function of probe delay for ZnO, Pd-ZnO, and PdZn-ZnO (probed at 520 nm, pumped at 365 nm). Time delay constants τ_1 and τ_2 referred to short-life and long-life spans were obtained by two-sided exponential fitting of the kinetic traces. The short-life span is derived from the process of trapping excitons in the defect state, while the long-life span reflects the recombination process of photogenerated charge carriers.[1,2]

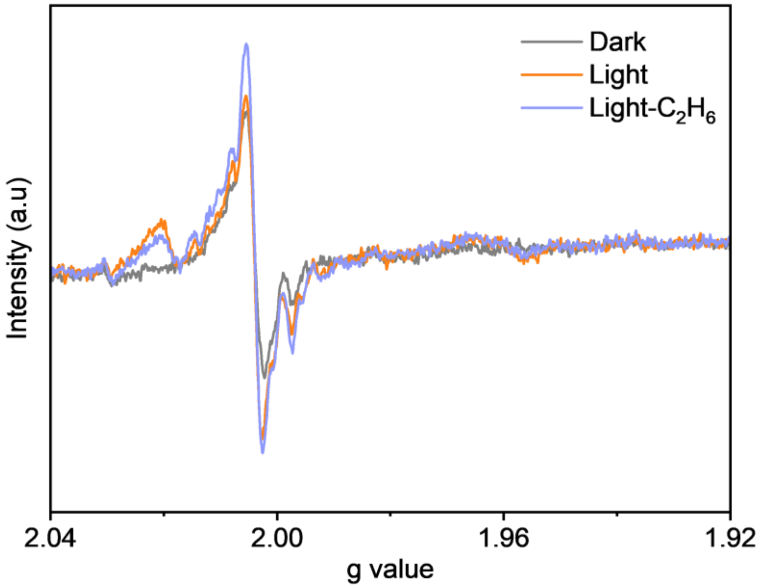

Supplementary Figure 32. In situ EPR spectra for PdZn-ZnO exposed to Ar in the dark and light conditions, as well as with exposure to C_2H_6 under light conditions. Irradiation condition: 365 nm LED, 600 mW cm^{-2}.

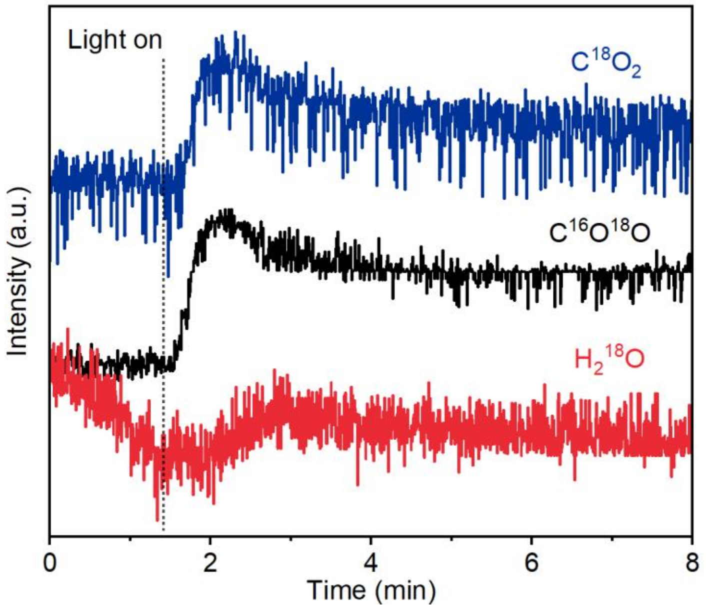

Supplementary Figure 33. MS spectra for $H_2{}^{18}O$, $C^{16}O^{18}O$, and $C^{18}O_2$ arising from photocatalytic ODHE with $^{16}O_2$ over PdZn-ZnO after ^{18}O exchange. PdZn-ZnO was first heated under a Ar + $H_2{}^{18}O$ flow at 300 °C for 3 h to incorporate ^{18}O into the ZnO lattice.[3-5] Photocatalytic ODHE was then performed using a C_2H_6 + $^{16}O_2$ feed gas. The presence of ^{18}O in the oxygen containing products of photocatalytic ODHE confirmed the participation of lattice oxygen from ZnO in the reaction.

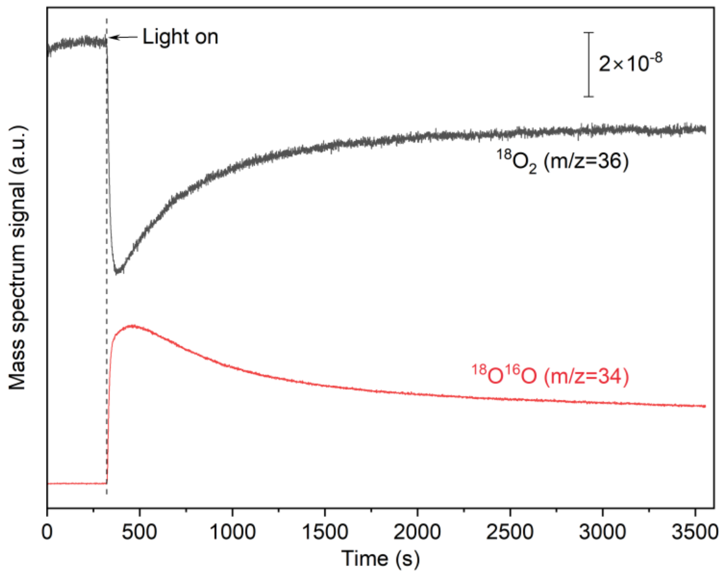

Supplementary Figure 34. Isotope exchange effect. Light-induced isotope exchange effect between $^{18}O_2$ and PdZn-ZnO.

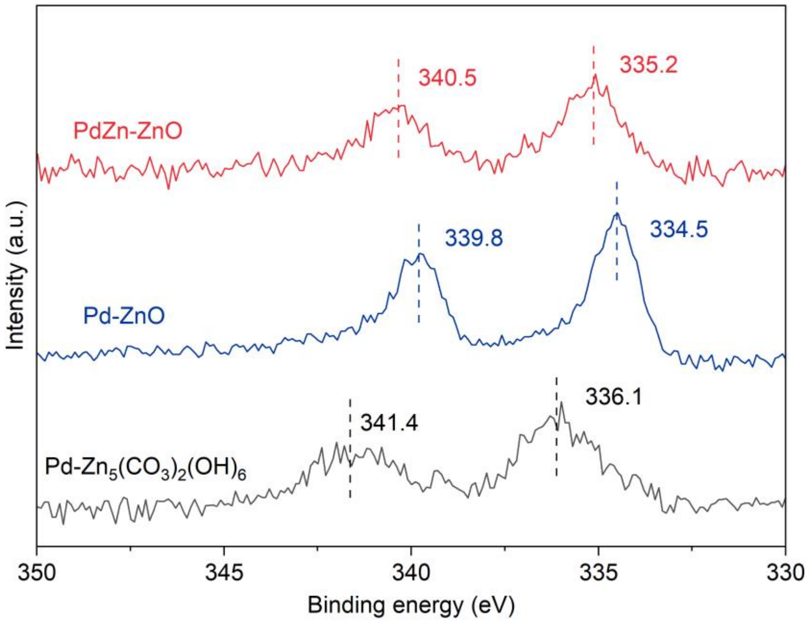

Supplementary Figure 35. Pd 3d XPS spectra for the PdZn-ZnO, Pd-ZnO, and Pd-$Zn_5(CO_3)_2(OH)_6$.

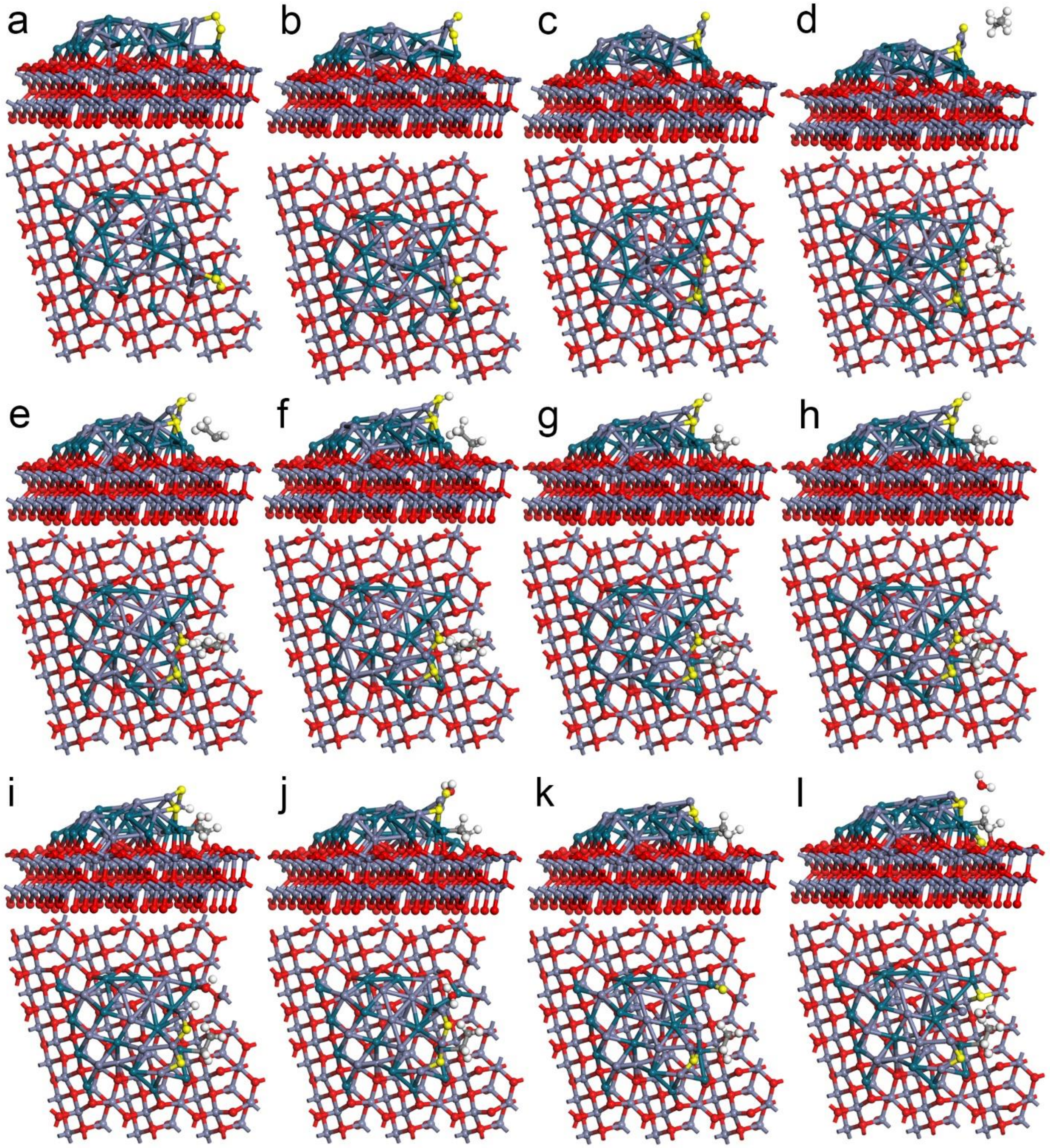

Supplementary Figure 36. Side and top views of optimized structures of reaction intermediates and TS on $Pd_{15}Zn_{15}$-ZnO. (a) *O_2. (b) TS1. (c) 2*O. (d) 2*O + *C_2H_6. (e) TS2. (f) *OH + *C_2H_5 + *O. (g) TS3. (h) *H + *OH + *C_2H_4 + *O. (i) TS4. (j) H_2O(g) + *C_2H_4 + 2*O + O_v. (k) TS5. (l) H_2O(g) + *C_2H_4 + *O. Red: O in ZnO, gray: Zn, white: H, yellow: O in O_2.

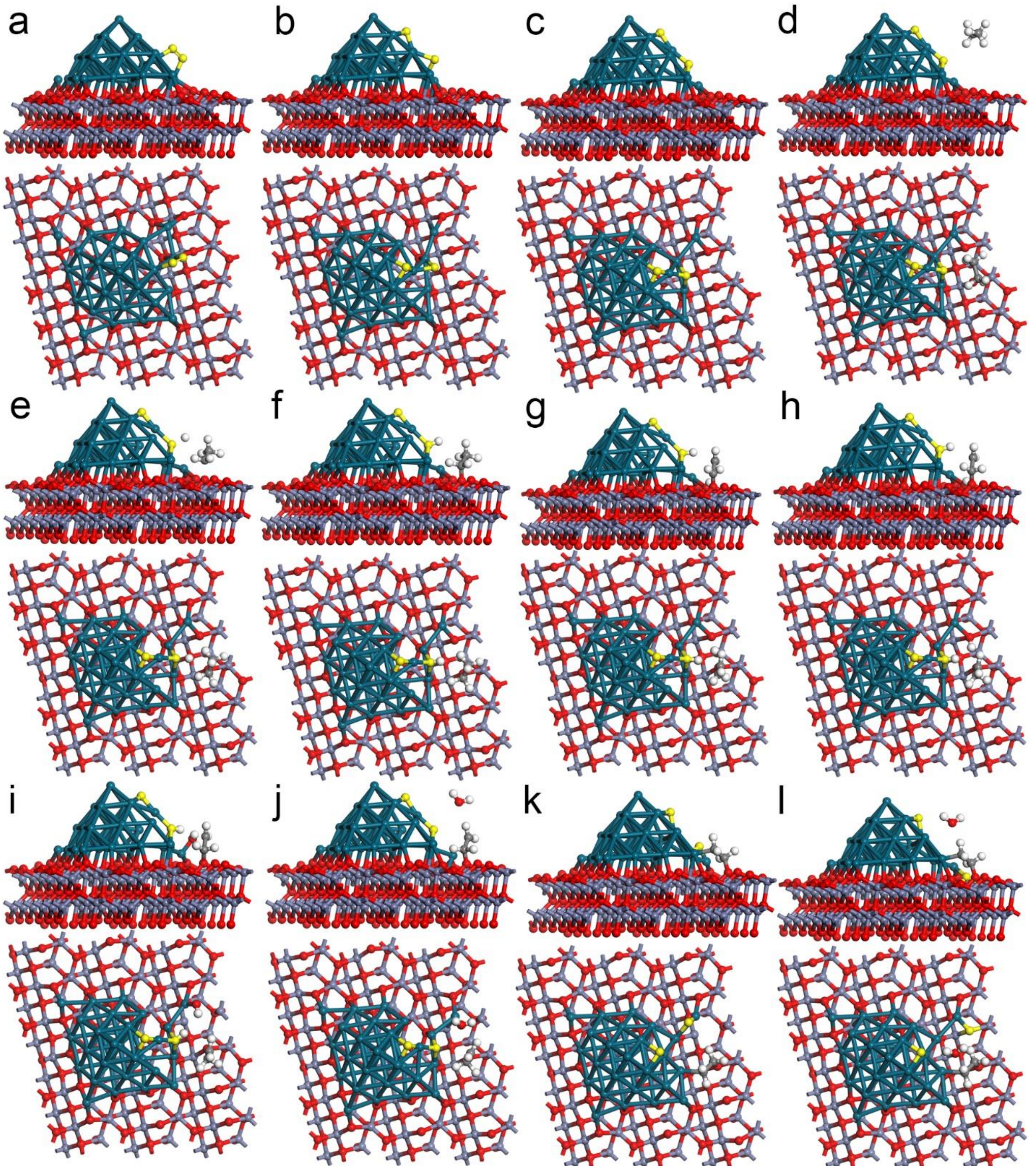

Supplementary Figure 37. Side and top views of optimized structures of reaction intermediates and TS on Pd$_{30}$-ZnO. (a) *O_2. (b) TS1. (c) 2*O. (d) 2*O + *C_2H_6. (e) TS2. (f) *OH + *C_2H_5 + *O. (g) TS3. (h) *H + *OH + *C_2H_4 + *O. (i) TS4. (j) H_2O(g) + *C_2H_4 + 2*O + O_v. (k) TS5. (l) H_2O(g) + *C_2H_4 + *O. Red: O in ZnO, gray: Zn, white: H, yellow: O in O_2.

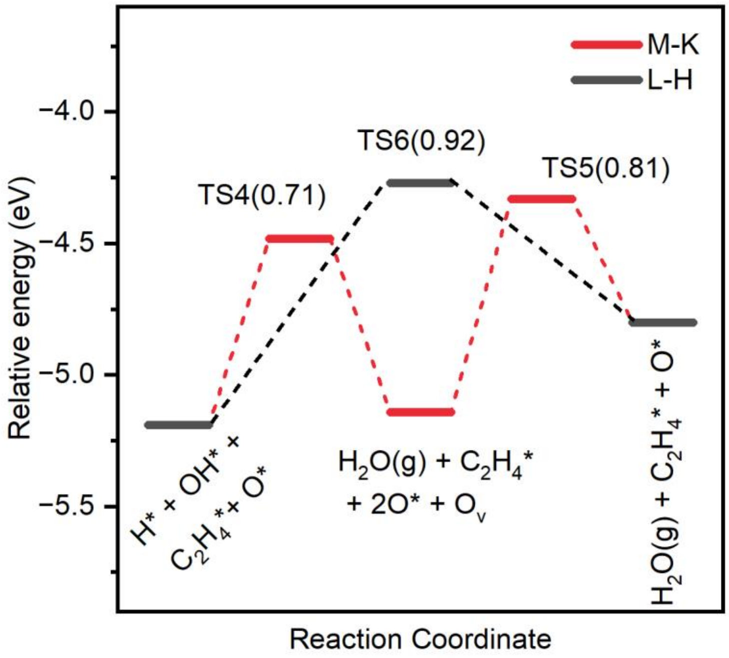

Supplementary Figure 38. Calculated potential energy diagrams for H_2O formation via M-K and L-H pathways on $Pd_{15}Zn_{15}$-ZnO.

M-K path: H_2O is formed by the reaction of adsorbed hydrogen atoms on the catalyst surface with active surface lattice oxygen in ZnO, leaving a surface oxygen vacancy which is then replenished by an oxygen atom from O_2 dissociation. L-H path: H_2O is formed by the reaction of adsorbed hydrogen atoms on the catalyst surface and a surface adsorbed oxygen atom (without lattice oxygen of ZnO being involved).

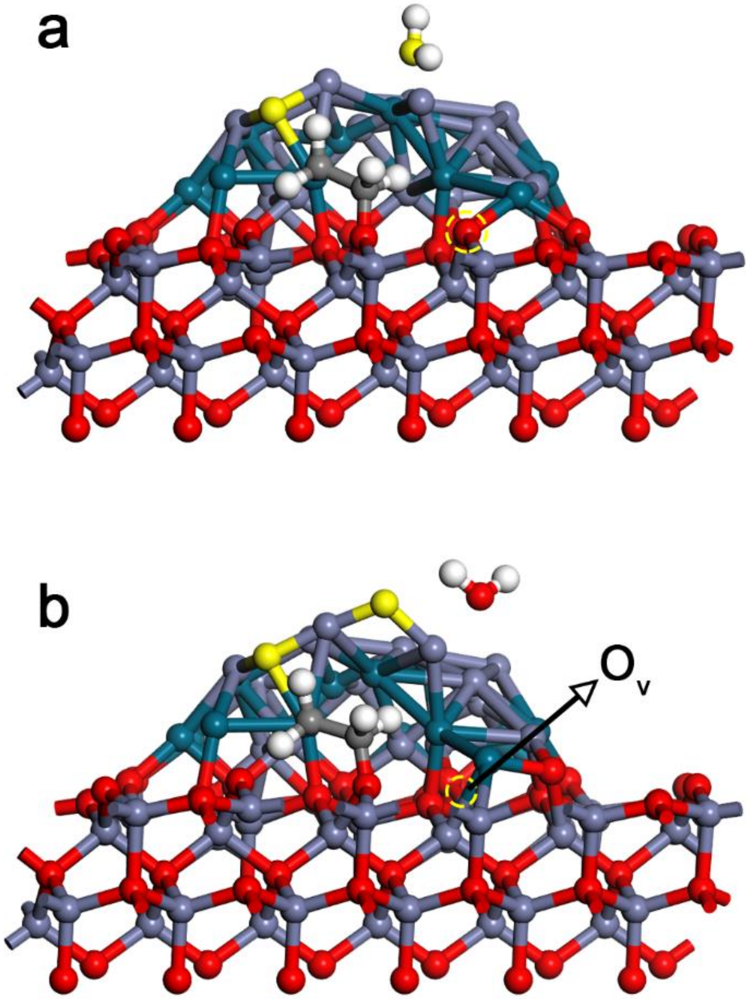

Supplementary Figure 39. Optimized structures for $Pd_{15}Zn_{15}$-ZnO. (a) H_2O(g) + *C_2H_4 + *O and (b) H_2O(g) + *C_2H_4 + 2*O + O_v on $Pd_{15}Zn_{15}$-ZnO. Red: O in ZnO, gray: Zn, white: H, yellow: O in O_2.

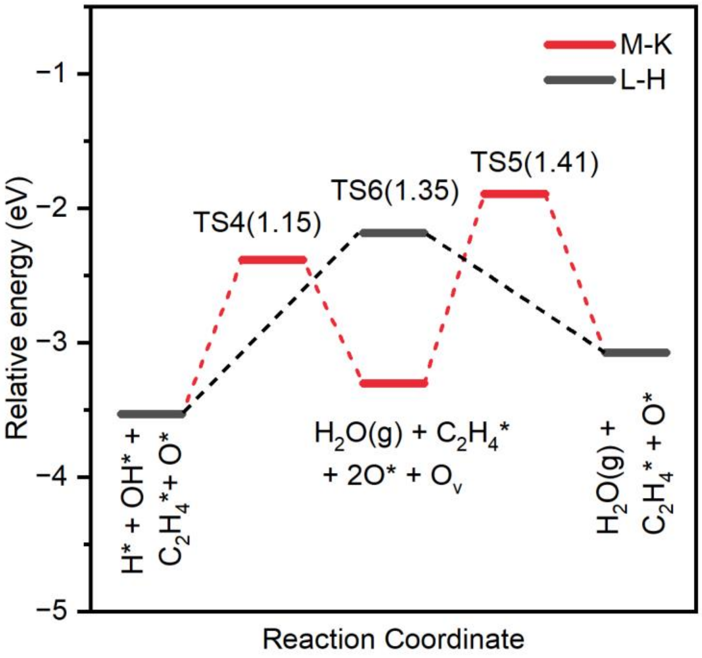

Supplementary Figure 40. Calculated potential energy diagrams for H_2O formation via M-K and L-H pathways on Pd_{30}-ZnO.

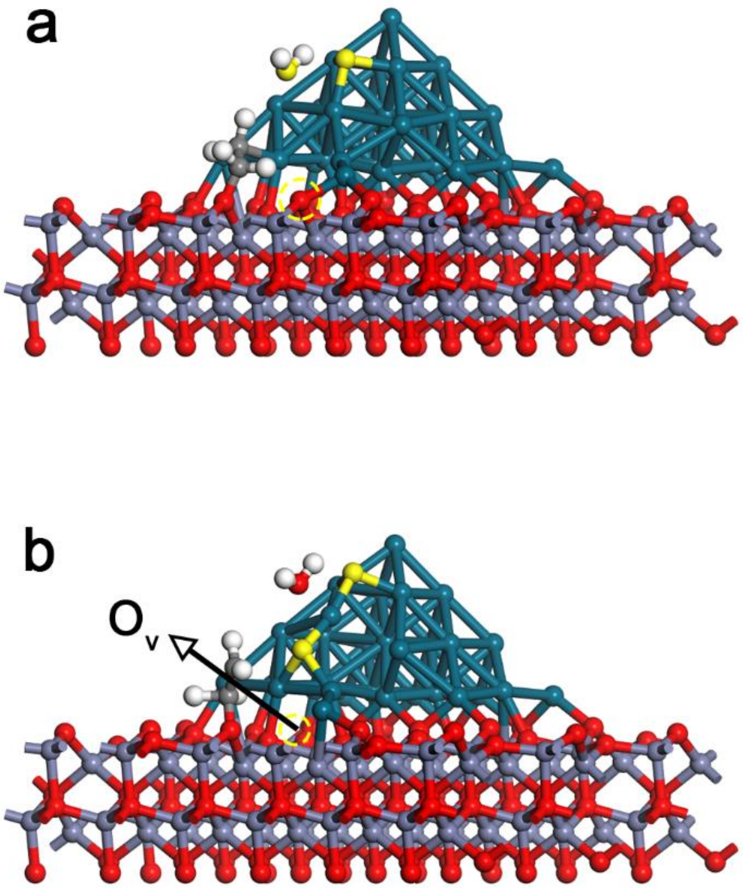

Supplementary Figure 41. Optimized structures for Pd_{30}-ZnO. (a) $H_2O(g)$ + *C_2H_4 + *O and (b) $H_2O(g)$ + *C_2H_4 + 2*O + O_v on Pd_{30}-ZnO. Red: O in ZnO, gray: Zn, white: H, yellow: O in O_2.

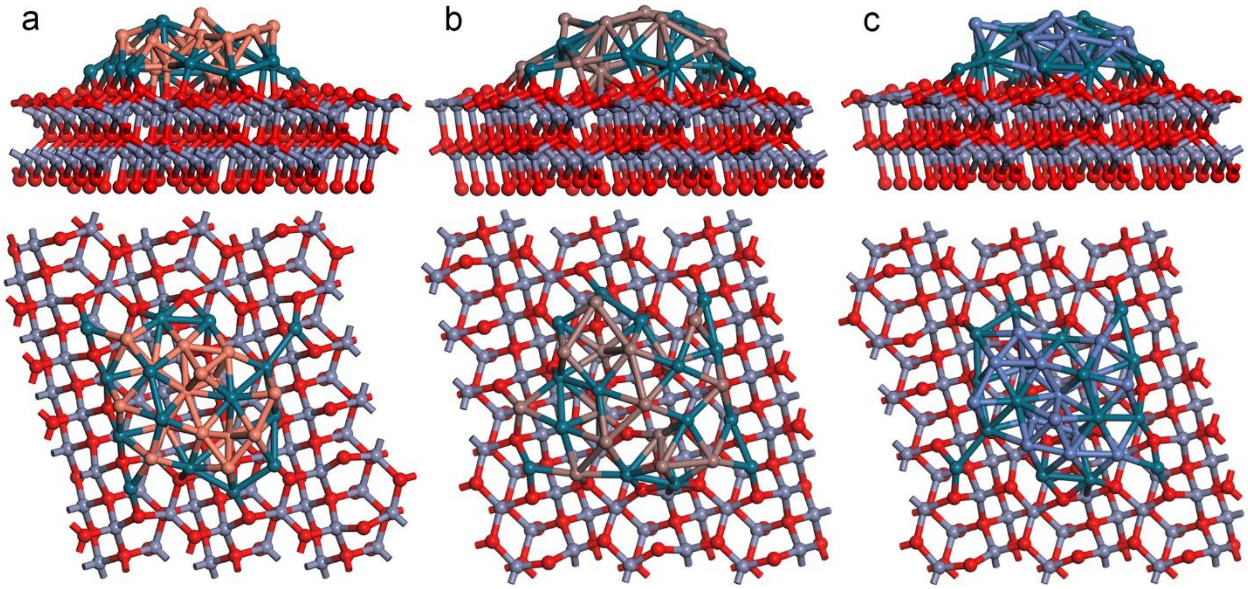

Supplementary Figure 42. Side and top views of optimized structures. (a) $Pd_{15}Cu_{15}$-ZnO, (b) $Pd_{15}In_{15}$-ZnO, and (c) $Pd_{15}Ni_{15}$-ZnO.

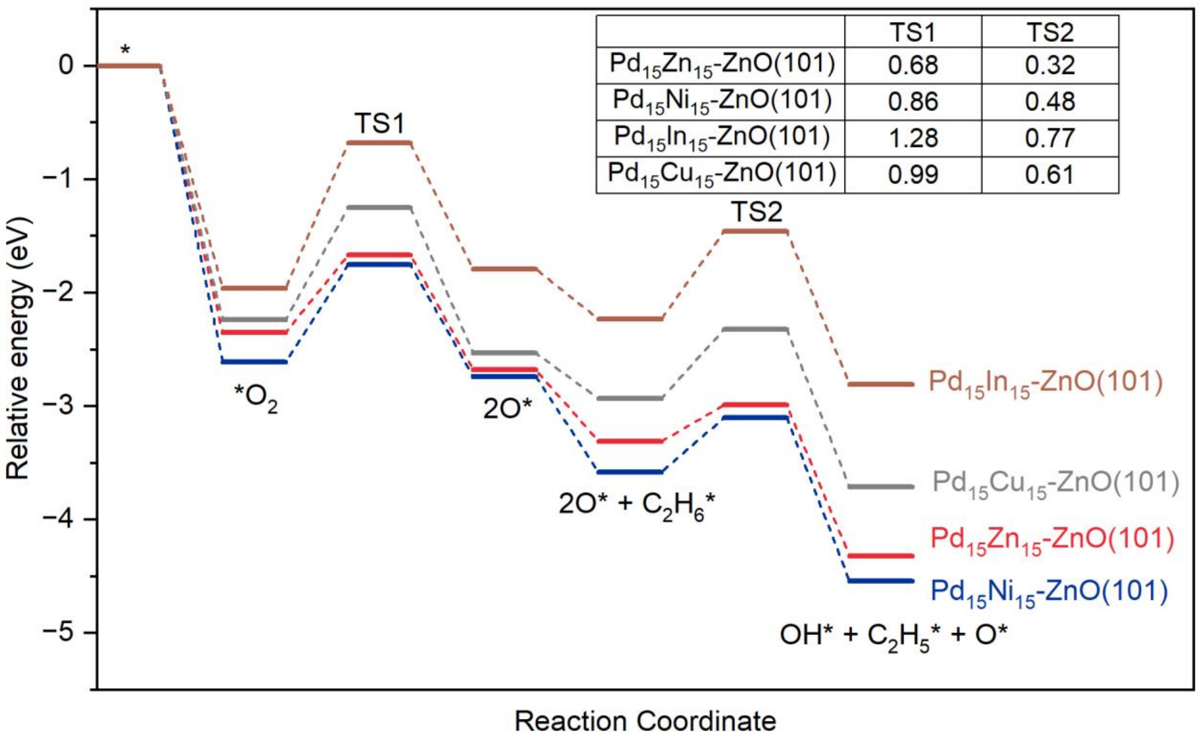

	TS1	TS2
$Pd_{15}Zn_{15}$-ZnO(101)	0.68	0.32
$Pd_{15}Ni_{15}$-ZnO(101)	0.86	0.48
$Pd_{15}In_{15}$-ZnO(101)	1.28	0.77
$Pd_{15}Cu_{15}$-ZnO(101)	0.99	0.61

Supplementary Figure 43. Calculated potential energy diagrams for TS1 and TS2 on $Pd_{15}In_{15}$-ZnO, $Pd_{15}Cu_{15}$-ZnO, $Pd_{15}Zn_{15}$-ZnO, and $Pd_{15}Ni_{15}$-ZnO.

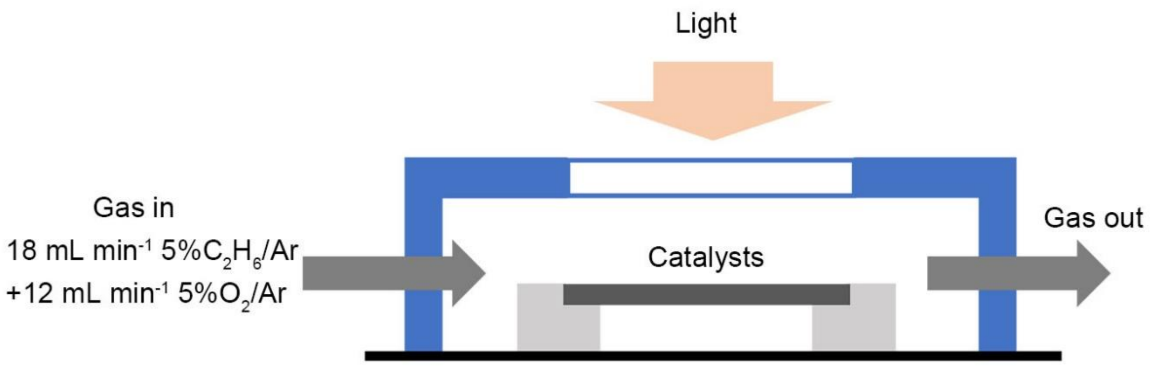

Supplementary Figure 44. Schematic diagram of the continuous flow photoreactor.

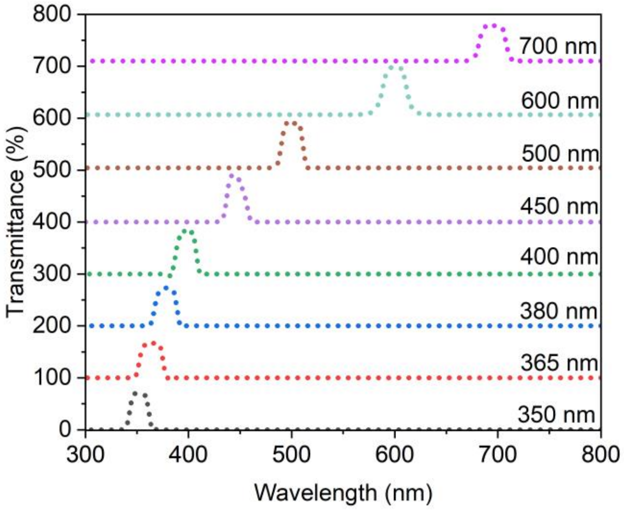

Supplementary Figure 45. Transmittance spectra of different wavelength filters.

Supplementary Table 1. Metal contents in different samples determined by ICP-OES.

**No.**	**Samples**	**Theoretical values (wt.%)**	**Measured values by ICP-OES (wt.%)**
1	0.1 wt.% PdZn-ZnO	0.2	0.06
2	0.5 wt.% PdZn-ZnO	0.5	0.31
3	1 wt.% PdZn-ZnO	1	0.75
4	2 wt.% PdZn-ZnO	2	1.57
5	3 wt.% PdZn-ZnO	3	2.90
6	2 wt.% Pd-ZnO	2	1.64
7	AuZn-ZnO	2	1.93
8	PtZn-ZnO	2	1.86
9	AgZn-ZnO	2	1.97
10	CuZn-ZnO	2	1.66
11	PdZn-TiO_2	2	1.41
12	PdZn-CeO_2	2	1.60
13	PdZn-In_2O_3	2	1.67
14	PdZn-Al_2O_3	2	2.06

Samples 1-6 and 11-14 contained Pd. Samples 7, 8, 9, and 10 contained Au, Pt, Ag and Cu, respectively.

Supplementary Table 2. Summarized data from the photocatalytic ODHE tests. Reaction conditions: 5.0 mg photocatalyst, C_2H_6 (5 vol% in Ar, 18 mL min^{-1}) + O_2 (1 vol% in Ar, 12 mL min^{-1}), 365 nm LED, 600 mW cm^{-2}.

**Catalyst**	**Amount of product (μmol g^{-1} h^{-1})**			**C_2H_4 selectivity (%)**	**C_2H_6 conversion (%)**
	**C_2H_4**	**CH_4**	**CO_2**		
PdZn-ZnO	46388	1766	5771	92.6	13.1
Pd-ZnO	15597	1509	4813	83.4	4.9
ZnO	171	334	4577	6.5	0.7
AgZn-ZnO	391	638	9225	7.3	1.4
AuZn-ZnO	256	68	2608	16.2	0.4
PtZn-ZnO	902	551	6969	9.3	1.2
CuZn-ZnO	766	946	5937	18.1	1.1
PdZn-TiO_2	13681	7030	23124	47.6	7.5
PdZn-In_2O_3	495	46	2688	26.5	0.5
PdZn-CeO_2	235	34	4033	11.4	0.6
PdZn-Al_2O_3	0	0	0	–	0
PdZn-ZnO-mix	1047	544	11307	15.2	1.9

Supplementary Table 3. Performance comparison of the PdZn-ZnO photocatalyst and other various recently reported thermocatalysts for ODHE.

No.	Catalyst	Reaction conditions	Space velocity ($L\ g^{-1}\ h^{-1}$)	C_2H_6 conversion rate ($mmol\ g^{-1}\ h^{-1}$)	C_2H_4 production rate ($mmol\ g^{-1}\ h^{-1}$)	C_2H_4 selectivity (%)	Ref.
**1**	PdZn-ZnO	365 nm LED, 600 mW cm^{-2}, 136 °C	360	50.1	46.4	92.6	This work
**2**	Pd-ZnO	365 nm LED, 600 mW cm^{-2}, 136 °C	360	18.7	15.6	83.4	This work
**3**	s-BN	575 °C	30	19.1	11.4	60	[6]
**4**	MoO_3-TiO_2	550 °C	10	22.5	20.7	92.1	[7]
**5**	5 wt.% $NaNO_3$-$Sr_{0.8}Ca_{0.2}FeO_{3-\delta}$	500 °C	6	28.7	26.1	91	[8]
**6**	NiO-CeO_2	275 °C	3	1.2	0.75	60.2	[9]
**7**	$MoVNbTeO_x/CeO_2$	400 °C	13.2	29.2	24.3	83.9	[10]
**8**	80Ni/75Ti-Si	400 °C	60	30.3	21.5	71	[10]
**9**	Nb_2O_5-NiO/Ni-foam	410 °C	9	22.9	15.4	68	[11]
**10**	45Al/Pd/Al_2O_3	675 °C	30	81.2	51.2	63	[12]
**11**	Mo-SBA-16	600 °C	4.4	13.7	8.2	60	[13]
**12**	Ni-Al-O	500 °C	6	12.1	9.1	75.3	[14]
**13**	$B_{0.1}CN$	520 °C	14.4	3.7	0.76	56	[15]
**14**	$MoVNbTeO_x$@Foam SiC	430 °C	7	27.2	37.5	91.4	[16]

Supplementary Table 4. Relative energy (*E*) for ODHE models on the $Pd_{15}Zn_{15}$-ZnO and Pd_{30}-ZnO surfaces.

**Model**	***E* (eV)**	
	$Pd_{15}Zn_{15}$-ZnO	Pd_{30}-ZnO
$*O_2$	–2.35	–1.54
TS1	–1.67	–0.46
2*O	–2.68	–1.72
2*O + $*C_2H_6$	–3.31	–1.89
TS2	–2.99	–1.03
*OH + $*C_2H_5$ + *O	–4.32	–2.48
TS3	–3.95	–2.19
*H + *OH + $*C_2H_4$ + *O	–5.19	–3.53
TS4	–4.48	–2.38
H_2O(g) + $*C_2H_4$ + 2*O + O_v	–5.14	–3.30
TS5	–4.33	–1.89
H_2O(g) + $*C_2H_4$ + *O	–4.80	–3.07
H_2O(g) + C_2H_4(g) + *O	–3.91	–2.21

Supplementary Table 5. E_a for ODHE models on the $Pd_{15}Zn_{15}$-ZnO and Pd_{30}-ZnO surfaces.

**Models**	**E_a (eV)**	
	$Pd_{15}Zn_{15}$-ZnO	Pd_{30}-ZnO
TS1	0.68	1.08
TS2	0.32	0.86
TS3	0.37	0.29
TS4	0.71	1.15
TS5	0.81	1.41
TS6	0.99	1.35

Supplementary References

1. Li, R. *et al.* Integration of an inorganic semiconductor with a metal–organic framework: a platform for enhanced gaseous photocatalytic reactions. *Adv. Mater.* **26**, 4783–4788 (2014).
2. Jiang, Y. *et al.* Elevating photooxidation of methane to formaldehyde via TiO_2 crystal phase engineering. *J. Am. Chem. Soc.* **144**, 15977–15987 (2022).
3. Shoji, S. *et al.* Photocatalytic uphill conversion of natural gas beyond the limitation of thermal reaction systems. *Nat. Catal.* **3**, 148-153 (2020).
4. Song, S. *et al.* A selective Au–ZnO/TiO_2 hybrid photocatalyst for oxidative coupling of methane to ethane with dioxygen. *Nat. Catal.* **4**, 1032–1042 (2021).
5. Watanabe, R. *et al.* Role of alkali metal in a highly active Pd/alkali/Fe_2O_3 catalyst for water gas shift reaction. *Appl. Catal. A–Gen.* **457**, 1–11 (2013).
6. Huang, R. *et al.* Direct insight into ethane oxidative dehydrogenation over boron nitrides. *ChemCatChem* **9**, 3293–3297 (2017).
7. Sarkar, B. *et al.* MoO_3 nanoclusters decorated on TiO_2 nanorods for oxidative dehydrogenation of ethane to ethylene. *Appl. Catal., B* **217**, 637–649 (2017).
8. Luongo, G. *et al.* Highly selective oxidative dehydrogenation of ethane to ethylene via chemical looping with oxygen uncoupling through structural engineering of the oxygen carrier. *Adv. Energy Mater.* **12**, 2200405 (2022).
9. Solsona, B., Concepción, P., Hernández, S., Demicol, B. & Nieto, J. M. L. Oxidative dehydrogenation of ethane over NiO–CeO_2 mixed oxides catalysts. *Catal. Today* **180**, 51–58 (2012).

10. Dang, D., Chen, X., Yan, B., Li, Y. & Cheng, Y. Catalytic performance of phase–pure M1 MoVNbTeO$_x$/CeO$_2$ composite for oxidative dehydrogenation of ethane. *J. Catal.* **365**, 238–248 (2018).

11. Zhang, Z. *et al.* Low–temperature, highly selective, highly stable Nb$_2$O$_5$–NiO/Ni–foam catalyst for the oxidative dehydrogenation of ethane. *Catal. Sci. Technol.* **8**, 4383–4389 (2018).

12. Lu, J. *et al.* Coking–and sintering–resistant palladium catalysts achieved through atomic layer deposition. *Science* **335**, 1205–1208 (2012).

13. Kong, L. *et al.* Oxidative dehydrogenation of ethane to ethylene over Mo–incorporated mesoporous SBA–16 catalysts: The effect of MoO$_x$ dispersion. *Appl. Catal. A–Gen.* **510**, 84–97 (2016).

14. Kong, L. *et al.* Template–induced mesoporous Ni–Al oxide catalysts with tuned physico–chemical properties for the oxidative dehydrogenation of ethane. *Chem. Eng. J.* **452**, 139247 (2023).

15. Cao, X. *et al.* Boron and nitrogen co–doped porous carbon nanospheres for oxidative dehydrogenation of ethane to ethylene. *Carbon* **197**, 120–128 (2022).

16. Yan, P., Chen, Y. & Cheng, Y. Industrially potential MoVNbTeOx@FoamSiC structured catalyst for oxidative dehydrogenation of ethane. *Chem. Eng. J.* **427**, 131813 (2022).
